# Multi-purpose cash transfers and health among vulnerable Syrian refugees in Lebanon: a prospective cohort study

**DOI:** 10.1186/s12889-021-11196-8

**Published:** 2021-06-19

**Authors:** Emily Lyles, Jakob Arhem, Ghada El Khoury, Antonio Trujillo, Paul Spiegel, Ann Burton, Shannon Doocy

**Affiliations:** 1 Department of International Health, Johns Hopkins Bloomberg School of Public Health, 615 N. Wolfe Street Suite E8132, Baltimore, MD 21205 USA; 2 United Nations High Commissioner for Refugees, Beirut, Lebanon; 3 School of Pharmacy, Lebanese American University, Byblos, Lebanon; 4 United Nations High Commissioner for Refugees, Geneva, Switzerland

**Keywords:** Cash transfers, Multi-purpose cash, Humanitarian assistance, Health, Syrian refugees, Lebanon

## Abstract

**Background:**

Multipurpose cash transfers (MPCs) are used on a widespread basis in the Syrian refugee response; however, there is little to no evidence as to how they affect health in humanitarian crises.

**Methods:**

A prospective cohort study was conducted from May 2018 through July 2019 to evaluate the impact of MPCs on health care-seeking and expenditures for child, adult acute, and adult chronic illness by Syrian refugees in Lebanon. Households receiving MPCs from UNHCR were compared to control households not receiving UNHCR MPCs.

**Results:**

Care-seeking for childhood illness was consistently high in both MPC and non-MPC households. An increased proportion of households did not receive all recommended care due to cost; this increase was 19.3% greater among MPC recipients than controls (*P* = 0.002). Increases in child hospitalizations were significantly smaller among MPC recipients than controls (DiD -6.1%; *P* = 0.037).

For adult acute illnesses, care-seeking increased among MPC recipients but decreased in controls (adjusted DiD 11.3%; *P* = 0.057); differences in change for other utilization outcomes were not significant. The adjusted difference in change in the proportion of MPC households not receiving recommended chronic illness care due to cost compared to controls was − 28.2% (*P* = 0.073). Access to medication for adult chronic illness also marginally significantly improved for MPC households relative to controls. The proportion of MPC recipients reporting expenses for the most recent child and adult acute illness increased significantly, as did the [log] total visit cost.

Both MPC and control households reported significant increases in borrowing to pay for health expenses over the year study period, but differences in change in borrowing or asset sales were not significant, indicating that MPC was not protective against for household financial risks associated with health.

**Conclusions:**

While MPC may have shown some positive effects, findings were mixed and MPC appears insufficient on its own to address health utilization and expenditures. A broader strategy addressing Syrian refugee health in Lebanon is needed of which MPC should be incorporated, with additional support such as additional conditional cash transfers for health.

**Supplementary Information:**

The online version contains supplementary material available at 10.1186/s12889-021-11196-8.

## Background

More than 5.5 million Syrian refugees are registered with the United Nations High Commissioner for Refugees (UNHCR) in neighboring countries throughout the region [1]. As of January 2020, nearly 1 million were registered in Lebanon, though the government estimates place the actual number closer to 1.5 million, making Lebanon host to the highest concentration of refugees per capita worldwide [2]. Syrian refugees live in informal settlements or integrated into Lebanese communities, with the largest number concentrated in the Bekaa valley [3].

Syrian refugees access services in existing Lebanese systems, which receive both technical and financial support as part of the humanitarian response [3]. The health sector employs a strategy whereby care is provided for both registered and unregistered Syrian refugees at subsidized rates (US$2–3) at more than 100 primary care facilities throughout Lebanon [3]. Secondary and tertiary health care for refugees is supported through a UNHCR cost-sharing program. The format of this program has changed over time, adapting to changed circumstances and available resources. Until 30 June 2018 the UNHCR hospital care program covered 75–90% of a hospital bill for an individual refugee [4]. As of 1st July 2018, a revised scheme was implemented in which the UNHCR introduced a threshold for support: hospital costs under US$100 would no longer be covered. Simultaneously, a cap was introduced to ensure that no refugee would pay more than US$800 for one single episode of hospital care in an effort to prevent catastrophic out of pocket expenditure for refugee households [5]. The changes were introduced to encourage utilization of primary health care centers for less severe episodes of acute illness rather than seeking care at hospital Emergency Rooms, thus, influencing health utilization patterns, health expenditures and their impact on household financial stability.

In addition to sector-specific responses, cash assistance is provided to Syrian refugees in Lebanon, reflecting global assistance trends where there is a shift away from in-kind assistance where feasible [6, 7]. Increasing routine use of cash programming is among the commitments made by humanitarian donors and organizations through the Grand Bargain at the 2016 World Humanitarian Summit and it is consequently among the most active Grand Bargain workstreams [8, 9]. Approximately US$5.6 billion in humanitarian assistance was disbursed through cash and voucher assistance (CVA) in 2019, a twofold increase from 2015 [10]. In 2019, the World Food Program (WFP) provided $2.1 billion in CVA, a 23% increase over 2018, while in the same period, UNHCR scaled up CVA from US$568 million to US$650 million (14% increase) [8, 10].

In Lebanon, cash assistance accounted for US$455 million, or 32% of the total Lebanon Crisis Response Plan (LCRP) appeal in 2019 and US$473 million (34% of the total LCRP appeal) in 2018, compared to only US$133 million (of US$1 billion total) in 2013 [11, 12]. The bulk of cash assistance for Syrian refugees in Lebanon is provided by UNHCR and WFP, primarily in the form of multipurpose cash transfers (MPCs) or unrestricted cash. UNHCR provides a monthly cash transfer of 260,000 LBP/US$173.5 to the most economically vulnerable families. Another group receives US$173.5 per family in MPC and US$27 per person for food from WFP. A third group^1^ receives US$27 per person for food from WFP, with some of these households also receiving the aforementioned UNHCR transfers [13].

There have been many claims regarding cash transfers, particularly MPCs, stating they are more efficient and effective than in-kind assistance, improve local economies, and provide more choice and dignity for affected persons; however, despite substantial evidence from development contexts, there is little to no evidence as to how MPCs affect health in humanitarian crises [14–17]. While cash can remove financial access barriers, potential impact on health indicators depends on a complex range of factors including cash transfer modality, conditionality and targeting; health policy and barriers to health access; and overall population health status. In the absence of well-designed research that assesses the effectiveness of MPCs on health in refugee settings, this study examined the effects of MPCs on health-seeking behavior, health service utilization, and health expenditures to provide much-needed evidence to inform use of cash transfer programs in both the current and future humanitarian responses.

## Methods

A prospective cohort study was conducted from May 2018 through July 2019 to evaluate the effectiveness of MPCs provided by UNHCR to vulnerable Syrian refugee households in increasing access to health. Households systematically sampled from UNHCR registration lists receiving MPCs from UNHCR at the start of this study (intervention group) and similarly vulnerable households not receiving MPCs from UNHCR and/or WFP^2^ (control group) were followed for 1 year to compare health expenditures, health-seeking behavior, and health service utilization. A parallel study in Jordan was also conducted in the same timeframe [Lyles E, Chua S, Barham Y, Trujillo A, Jardenah D, Spiegel P, et al: Multi-purpose cash transfers and health among vulnerable Syrian refugees in Jordan: a prospective cohort study, Manuscript under review].

### Outcome measures

The primary outcomes of interest in this study included health service utilization for the most recent household illness (es), specifically, the prevalence of health care-seeking, ability to obtain prescribed medicine(s), care visit type (i.e., inpatient, outpatient, or emergency room), and needed care averted due to cost. Additionally, out-of-pocket expenditures for care received for the most recent illness (es) were evaluated, including health facility payments for outpatient care and medications purchased at pharmacies outside the health facility where care was sought. These indicators were evaluated for the most recent household illnesses in each of three categories: childhood illness, adult acute illness, and adult chronic illness. Routine spending on health was also assessed, specifically, total household health expenditures in the preceding month as well as the prevalence of selling assets and borrowing money to pay for health expenses in the past 3 months.

### Sampling

Funding constraints limited the number of households that received MPC assistance, such that at the time of study initiation 53,016 or 24.3% of all refugee households received MPCs from either UNHCR or WFP; a total of 124,961 refugee households were classified by UNHCR as severely vulnerable, thus only 42.4% of households eligible for MPCs were receiving them (see Additional file 1 for more details). The result, where households with similar levels of economic vulnerability did or did not receive MPCs, allowed for the examination of health outcomes associated with cash assistance in what are perceived as two comparable groups of households. To reduce variability between comparison groups and the risk of cross-over due to changes in targeting methods and anticipated scale-up of MPC programs, the sample was restricted to households with estimated per capita monthly expenditures between US$60–70 as these were believed to be less likely to be impacted by planned changes to targeting criteria during the intervention period.

Sample size calculations were based on the primary research aim of comparing vulnerable households receiving MPCs to similar households not receiving MPCs. Most outcome measures of interest (e.g., care-seeking or having out-of-pocket health expenditures) can be expressed as a proportion. Calculations assumed the most conservative proportion of 50%,^3^ power = 0.80, a minimum detectable difference of ≥10%, and were two-sided. Based on these assumptions, a minimum required sample size of 770 households (385 per group) was identified; this was increased to a minimum planned sample of 1000 households to allow for loss to follow-up of ≤30%. The sample was allocated by region proportionally to the location of UNHCR MPC beneficiaries with similar numbers in the intervention and control groups. The number of non-MPC households in Bekaa was increased in anticipation of crossover of some households due to planned expansion of MPCs by WFP. Within each region, lists of MPC recipients and non-MPC recipients were ordered by estimated per capita expenditure and systematically sampled. Sampling lists included twice as many households as the projected sample from each region to allow for households that were unreachable, declined to participate, or were determined to be ineligible during screening questions ahead of enrollment interviews. Enrollment continued until the target sample size (*n* = 1000) was reached. Enumerators attempted to reach a total of 2102 households for study enrollment, of which 71 (3.4%) declined to participate in the study, 966 (46.0%) could not be reached, and 60 (2.9%) were deemed ineligible, most commonly because they were no longer in Lebanon. A total of 1005 households enrolled in the study, 898 (89.4%) of which were also reached at endline.

UNHCR and WFP revise the list of MPC recipients annually to reflect changes in funding and households’ financial situations to ensure households currently deemed most economically vulnerable are targeted. In November 2018, following recalibration of the desk formula used to determine refugee households’ vulnerability level, some existing beneficiaries stopped receiving MPCs whilst others not previously receiving MPCs were added to the MPC beneficiary list. This resulted in many (*n* = 388; 78.1%) study participants enrolled as MPC beneficiaries transitioning off UNHCR MPCs for the second half of the study while fewer participants (*n* = 64; 12.6%) enrolled in the control group began receiving UNHCR MPCs. To maximize power in analyses given reduced sample sizes resulting from the recalibration, participants receiving MPCs from UNHCR at endline (i.e., those who received MPCs for the entire study period and those who began receiving MPCs at the study mid-point) were analyzed as MPC beneficiary households (intervention group) while the control group included only those not receiving MPCs from UNHCR through the entire study period. The sample at enrollment and the final analyzed sample are presented in Table 1; reasons for loss to follow-up are provided in Additional file 1 and detail on change in intervention receipt is presented in Additional file 1, Table 2.

**Table 1:** Enrolled Sample and Analyzed Sample by Region

Region	UNHCR MPC Recipients by Region at Enrollment	Enrolled Sample (***n*** = 1005)				Analyzed Sample (***n*** = 617)			
		Intervention (***n*** = 497)		Control (***n*** = 508)		Intervention (***n*** = 173)		Control (***n*** = 444)	
		N	%	N	%	N	%	N	%
North	23.0%	112	22.5%	109	21.5%	71	41.0%	89	20.0%
BML	12.5%	65	13.1%	50	9.8%	20	11.6%	44	9.9%
Bekaa	58.0%	278	55.9%	306	60.2%	60	34.7%	279	62.8%
South	6.4%	42	8.5%	43	8.5%	22	12.7%	32	7.2%

**Table 2:** Sample Demographic Characteristics and Living Conditions at Baseline and Endline

	BASELINE					ENDLINE				
	MPC HHs (***N*** = 173)		Control HHs (***N*** = 444)		*P* value	MPC HHs (***N*** = 168)		Control HHs (***N*** = 375)		*P* value
**Principal Applicant/Household Head Characteristics**										
Female sex	68	(39.3%)	212	(47.7%)	0.059	65	(38.7%)	175	(46.7%)	0.084
Age (mean years)	39.7	(9.0)	38.0	(12.1)	0.101	40.9	9.2	38.8	11.8	**0.041**
Highest level of education										
None	52	(30.2%)	101	(22.9%)	**0.007**	46	(27.4%)	66	(17.6%)	**0.001**
Primary school	91	(52.9%)	215	(48.8%)		100	(59.5%)	207	(55.3%)	
Preparatory school	22	(12.8%)	72	(16.3%)		16	(9.5%)	56	(15.0%)	
Secondary school	7	(4.1%)	53	(12.0%)		6	(3.6%)	45	(12.0%)	
Marital status										
Married	155	(89.6%)	408	(91.9%)	0.248	150	(89.3%)	337	(89.9%)	0.984
Widowed	6	(3.5%)	21	(4.7%)		6	(3.6%)	11	(2.9%)	
Never married / Divorced	4	(2.3%)	5	(1.1%)		3	(1.8%)	7	(1.9%)	
**Household Demographic Characteristics**										
Household size (mean)	7.6	(2.5)	6.0	(2.4)	***< 0.001***	7.5	2.5	6.0	2.1	***< 0.001***
Dependency ratio ^a^ (mean)	2.3	(1.0)	1.7	(1.0)	***< 0.001***	2.4	1.2	1.7	1.1	***< 0.001***
Multiple UNHCR registration cases (%)	5	(2.9%)	11	(2.5%)	0.772	9	(5.4%)	31	(8.3%)	0.230
Vulnerable members (%)										
Child(ren) < 5 yrs	136	(78.6%)	317	(71.4%)	0.068	111	(66.1%)	250	(66.7%)	0.892
Child(ren) ≤ 17 yrs	172	(99.4%)	426	(95.9%)	**0.025**	166	(98.8%)	364	(97.1%)	0.219
Older adult(s) (> 60 yrs)	16	(9.2%)	51	(11.5%)	0.422	20	(11.9%)	53	(14.1%)	0.482
Member with a chronic health condition	27	(15.6%)	66	(14.9%)	0.817	60	(35.7%)	136	(36.3%)	0.901
Member w/ disability or that needs daily support	23	(13.3%)	46	(10.4%)	0.299	45	(26.8%)	68	(18.1%)	**0.022**
**Living Conditions**										
Residence type										
Apartment or house	61	(35.3%)	211	(47.5%)	**0.006**	65	(38.7%)	142	(37.9%)	0.292
Single room	79	(45.7%)	159	(35.8%)		73	(43.5%)	149	(39.7%)	
Temporary shelter ^b^	21	(12.1%)	61	(13.7%)		16	(9.5%)	58	(15.5%)	
Other ^c^	12	(6.9%)	13	(2.9%)		14	(8.3%)	26	(6.9%)	
Residence arrangement										
Rented	159	(91.9%)	401	(90.3%)	0.206	156	(92.9%)	345	(92.0%)	0.672
Hosted for free / rent paid by NGO/charity	12	(6.9%)	42	(9.5%)		8	(4.8%)	22	(5.9%)	
Owned	2	(1.2%)	1	(0.2%)		4	(2.4%)	6	(1.6%)	
Shared living space	156	(90.7%)	383	(86.3%)	0.135	167	(99.4%)	375	(100%)	0.135
Crowding (mean # people/sleeping room)	4.5	(1.9)	3.6	(1.7)	***< 0.001***	4.3	2.0	4.1	1.9	0.228

Presented as N (%) or mean (standard deviation). Bold italic indicates statistically significant (p < 0.001) findings; bold indicates statistically significant (*p* < 0.05) findings; italic indicates statistically significant (*p* < 0.10) findings

^a^ Number of dependents divided by number of working age adults

^**b**^ includes tent, prefab unit, collective center

^c^ includes unfinished building, construction site, factory, or warehouse

The final analyzed sample (*N* = 617) included households that were receiving MPCs from UNHCR at endline as MPC beneficiary households (i.e., intervention group, *n* = 173) and control households consisting of only those not receiving MPCs for the study’s duration (*n* = 444). Of these 617 enrolled households, 168 (97.1%) MPC households and 375 (84.5%) control households were followed for a one-year period and completed endline interviews.

### Study implementation

Potential participants were identified using lists of refugee households registered with UNHCR in Lebanon. Sampled households were contacted by phone using telephone numbers provided to UNHCR, screened to confirm eligibility, and invited to participate in the study. Household heads (as indicated in the UNHCR registration) served as preferred respondents; however, in cases where the individual did not regularly reside with the household or could not be reached, another adult household member served as the primary respondent. Households were deemed ineligible if they could not be reached after three phone calls or if an appropriate and willing respondent could not be reached.

Due to the use of phone interviews with no face-to-face contact with participants and given high levels of illiteracy in the Syrian population, oral informed consent was obtained for participation in the study prior to enrollment interviews; an abbreviated oral consent was used at endline to confirm agreement for continued participation. Use of oral informed consent was approved by the Johns Hopkins Bloomberg School of Public Health (JHSPH) and Lebanese American University Institutional Review Boards. Consenting households enrolled in the study completed phone interviews at the start of the study (May–July 2018) and after a one-year follow-up period (May–July 2019). Phone interviews have been used for health research by UNHCR and academic institutions in Lebanon, have acceptable response rates, and are considered contextually/culturally appropriate [18–20]. In the present study, phone interviews were preferable both for logistical and cost efficiency and for privacy protection, as compared to in-person interviews where neighbors could become aware of participation. Interviews lasted between 30 and 50 min and used a structured questionnaire (see Additional file 2) focused on household demographic and socioeconomic characteristics, receipt of humanitarian assistance, and household health seeking behavior, health service utilization, and health expenditures for child, adult acute, and chronic illnesses. Interviews were completed by Lebanese university students who received four half-days of classroom training on the questionnaire, e-data collection, interview techniques, and basic principles of human subjects’ protections followed by two half-days of supervised practice completing interviews. Data were collected on tablets using the Magpi mobile data platform by DataDyne LLC (Washington, DC). Interviewers were supervised by both a national study coordinator and the JHSPH study coordinator, with daily review of uploaded records to enable prompt identification of data quality concerns.

### Data analysis

Data analysis was performed using Stata 13 (College Station, TX) software. Differences in descriptive statistics between study groups (i.e. MPC vs. non-MPC beneficiaries) were examined using chi-square and t-test methods for binary/categorical and continuous variables, respectively. Regression models were used to evaluate the effects of MPCs on health outcomes, both unadjusted and controlling for differences in household characteristics. Linear probability models were used to estimate differences in binary outcomes between study groups from baseline to endline with main terms for study group (i.e., MPC or control), time period (i.e., baseline or endline), and the interaction between study group and time period. Log-linear models were similarly used to estimate differences in continuous outcomes; log transformation was required for health expenditure outcomes due to their skewed distribution. Coefficients for the interaction of study group and time period represent the estimated difference in change comparing MPC beneficiaries to non-beneficiaries (i.e., the difference-in-difference/“MPC effect”). Effect sizes associated with receipt of MPCs were also calculated by dividing difference-in-difference (DiD) by the overall mean for each respective outcome. All models utilized cluster-robust standard errors with clustering defined at the household level, allowing for correlation between baseline and endline observations for each household.

Financial indicators are presented in U.S. Dollars (US$)^4^ using an exchange rate of 1507.5 LBP/US$1 [21]. All variables reporting monetary values for income or expenditures were assessed for outliers using visual inspection and the general guidance that points falling three or more standard deviations from the mean should be considered as potential outliers. Outliers that appeared to be the result of misreporting or recording errors were corrected or removed from the data set. Other outliers were checked with field teams for accuracy and corrected as needed. Preliminary analysis and findings were discussed by all the collaborating organizations prior to finalization of results to ensure their accuracy and the best possible interpretation of findings within the Lebanese context.

The study was approved by the Institutional Review Boards at Lebanese American University and JHSPH.

## Results

### Study sample characteristics

Characteristics of the principal applicant, household composition, and living conditions are summarized in Table 2. At baseline, there were no significant differences in most principal applicant characteristics between MPC recipients and controls/non-recipients, though non-recipient principal applicants had significantly higher educational attainment; at endline, principal applicants in recipient households were significantly older and still comparably less educated than in non-recipient households. MPC households had significantly larger household size and dependency ratios, were more likely to have children, and had significantly different living conditions at baseline. At endline, recipient households were significantly larger with higher dependency ratios and disabled household members, but living conditions were similar between groups. Households’ economic characteristics (Table 3) differed significantly at baseline in terms of both income and expenditure, with MPC recipients reporting significantly greater incomes and expenditures. Mean incomes and expenditures increased from baseline to endline in both groups, though only expenditures differed significantly between groups at endline. Significant differences in receipt of assistance were observed at baseline and endline with respect to both the proportion of households receiving assistance and total amount received. Of note, the proportion of households receiving WFP assistance increased among MPC recipients but decreased among controls over the study period and WFP transitioned some households from e-vouchers to MPCs.

**Table 3:** Household Economy and Receipt of Humanitarian Assistance

	BASELINE					ENDLINE				
	MPC HHs (***N*** = 173)		Control HHs (***N*** = 444)		*P* value	MPC HHs (***N*** = 168)		Control HHs (***N*** = 375)		*P* value
**Household Income and Expenditures (past month; mean US$** ^**a**^**)**										
Income (excluding humanitarian assistance)	389	(427.9)	295	(354.4)	**0.005**	440	(628.9)	371	(390.8)	0.119
Total expenditures	693	(371.2)	553	(372.6)	***< 0.001***	778	(357.4)	695	(334.9)	**0.009**
**Total Humanitarian Assistance (past month)** ^**b**^										
**Any regular transfer (% of HHs)**	173	(86.1%)	444	(76.6%)	***0.009***	168	(86.9%)	375	(59.5%)	***< 0.001***
Amount received (mean US$^a^ per HH)	304	(109.8)	168	(92.5)	***< 0.001***	297	(141.2)	113	(117.8)	***< 0.001***
Amount received (mean US$^a^ per HH member)	42	(15.3)	30	(17.1)	***< 0.001***	43	(25.9)	20	(21.3)	***< 0.001***
**In-kind assistance (past 3 months)**	2	(1.2%)	10	(2.3%)	0.332	19	(11.3%)	47	(12.6%)	0.671
**WFP Food Assistance (past month)**										
Current WFP recipients	143	(82.7%)	334	(75.2%)	**0.048**	168	(85.7%)	375	(57.1%)	***< 0.001***
Amount received (mean US$^a^ per HH)	190	(62.6)	143	(70.7)	**< 0.001**	186	(68.1)	146	(48.0)	***< 0.001***
Amount received (mean US$^a^ per HH member)	26	(6.0)	25	(12.5)	0.519	25	(5.2)	25	(7.3)	0.424
Transfer modality										
E-Voucher	137	(95.8%)	312	(93.4%)	0.309	168	(100%)	367	(97.9%)	***0.056***
Multipurpose Cash Transfer	6	(4.2%)	22	(6.6%)		0	(0.0%)	8	(2.1%)	
**Asset Sales and Borrowing**										
Sold assets in past 3 months (%)	14	(8.1%)	42	(9.5%)	0.712	17	(10.1%)	74	(19.7%)	**0.006**
Borrowed money in past 3 months (%)	77	(44.5%)	166	(37.4%)	0.266	154	(91.7%)	331	(88.3%)	0.431
Current debt										
Any Debt	142	(86.1%)	321	(77.5%)	**0.021**	152	(92.7%)	326	(91.1%)	0.536
Amount of debt (among those w/ debt; mean US$^a^)	536	(628.8)	516	(674.3)	0.740	843	(1022.4)	711	(781.0)	0.106

Presented as N (%) or mean (standard deviation). Bold italic indicates statistically significant (*p* < 0.001) findings; bold indicates statistically significant (*p* < 0.05) findings; italic indicates statistically significant (*p* < 0.10) findings

^a^ Exchange rate: 1507.5 LBP = 1 US$

^b^ includes UNHCR, WFP, and regular monthly assistance from other less common sources

### Health service utilization

**Fig. 1:**
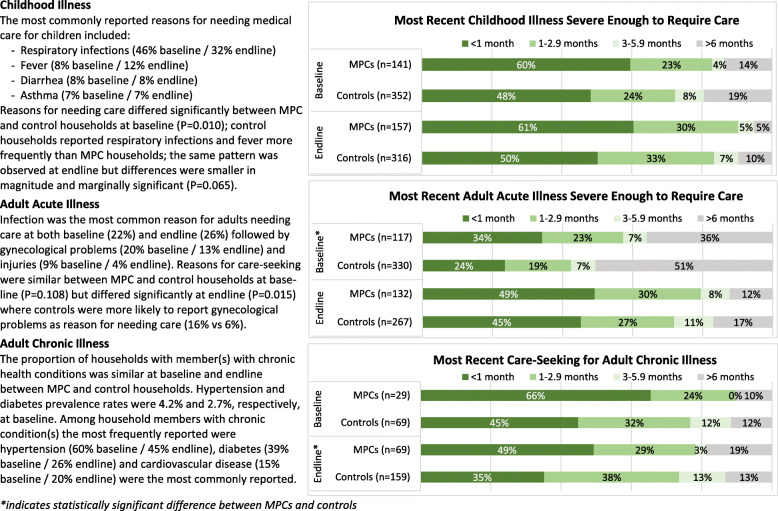
Reasons and Timeframes for Care-Seeking

**Fig. 2:**
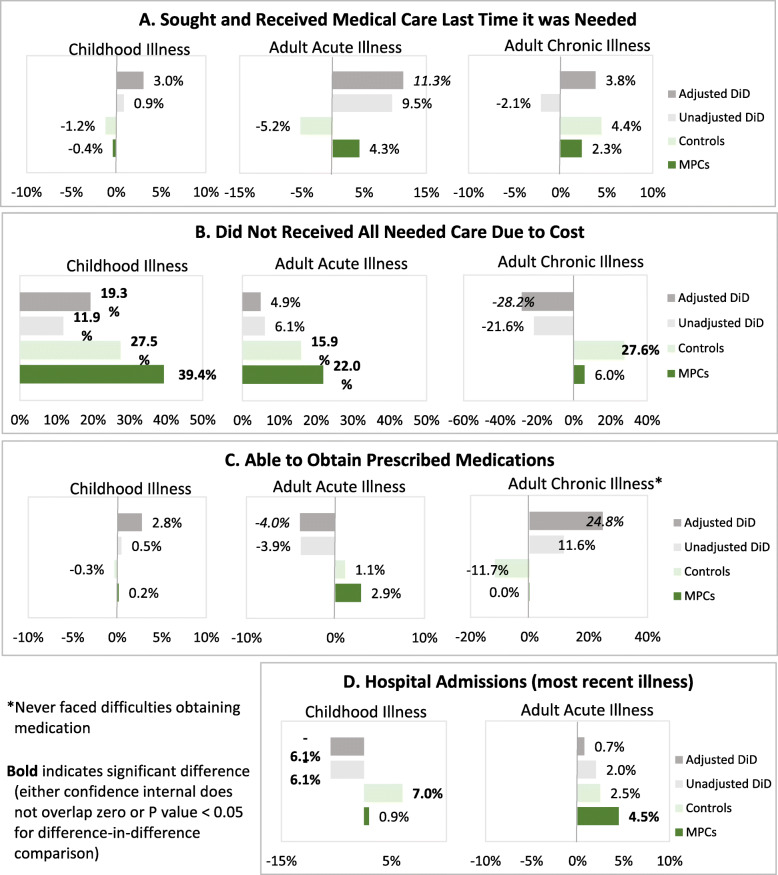
Change in Health Utilization Measures by Group and Difference in Magnitude of Change Between Groups

Care-seeking was evaluated for the most recent household member illness (within the past 6 months) that was perceived as severe enough to require medical care; results are reported for children and for acute and chronic illness among adults. Baseline and endline descriptive analyses by group are presented in Additional file 3 and Fig. 1; unadjusted and adjusted individual group change and differences in change between groups are provided in Table 4 and Fig. 2.

**Table 4:** Change in health care-seeking and medicines for household member illness

	UNADJUSTED CHANGE OVER TIME						ADJUSTED CHANGE OVER TIME ^**a**^								
	MPC HHs		Control HHs		DiD		MPC HHs		Control HHs		DiD ^**b**^		Overall %	Effect Size	R^**2**^
	%	(95% CI)	%	(95% CI)	%	(95% CI)	%	(95% CI)	%	(95% CI)	%	(95% CI)			
**Most Recent Childhood Illness**															
Sought and received medical care	-0.4%	(−3.6,2.8)	−1.2%	(−5.6,3.1)	0.9%	(−4.5,6.2)	0.7%	(−2.9,4.3)	−2.3%	(−7.4,2.7)	3.0%	(−2.9,9.0)	94.6%	3.2%	0.059
Able to obtain prescribed meds	0.2%	(−2.0,2.3)	−0.3%	(−2.4,1.7)	0.5%	(−2.5,3.4)	1.3%	(−0.6,3.3)	−1.4%	(−3.8,1.0)	2.8%	(−0.6,6.1)	98.4%	2.8%	0.029
Outpatient visit	**11.0%**	**(3.5,18.5)**	*5.6%*	*(−0.4,11.5)*	5.4%	(−4.2,15.0)	**11.1%**	**(2.8,19.3)**	6.7%	(−0.4,13.7)	4.4%	(−6.3,15.1)	87.4%	5.1%	0.032
Emergency room visit	***−11.9%***	***(− 18.5,-5.3)***	***− 12.6%***	***(− 17.6,-7.5)***	0.7%	(−7.7,9.0)	**− 12.3%**	**(− 19.4,-5.2)**	***−13.9%***	***(−20.0,-7.8)***	1.6%	(− 7.6,10.8)	8.1%	19.9%	0.071
Hospital admission	0.9%	(−2.6,4.4)	***7.0%***	***(3.6,10.3)***	**−6.1%**	**(−10.9,-1.2)**	1.2%	(−3.0,5.4)	***7.3%***	***(3.5,11.0)***	**− 6.1%**	**(− 11.7,-0.4)**	4.5%	−133.5%	0.043
Care not sought b/c of cost	−33.3%	(−90.6,23.9)	−11.4%	(−30.9,8.1)	−21.9%	(−82.4,38.5)	−53.3%	(− 143.1,36.4)	− 20.3%	(−44.6,4.0)	− 33.0%	(− 122.8,56.8)	87.7%	−37.7%	0.379
All care not received due to cost	***39.4%***	***(30.0,48.8)***	***27.5%***	***(20.7,34.4)***	**11.9%**	**(0.2,23.5)**	***39.8%***	***(30.6,49.1)***	***20.5%***	***(12.8,28.3)***	**19.3%**	**(7.3,31.2)**	24.6%	78.3%	0.177
**Most Recent Adult Acute Illness**															
Sought and received medical care	4.3%	(−4.1,12.6)	−5.2%	(−11.6,1.1)	*9.5%*	*(−1.0,20.0)*	6.9%	(−2.8,16.6)	−4.4%	(−10.8,2.1)	*11.3%*	*(−0.3,22.9)*	88.9%	12.7%	0.047
Able to obtain prescribed meds	*−2.9%*	*(−6.1,0.4)*	1.1%	(−1.8,3.9)	*−3.9%*	*(−8.2,0.4)*	*−4.0%*	*(− 8.2,0.3)*	0.0%	(−2.9,2.9)	*− 4.0%*	*(− 8.7,0.7)*	98.1%	− 4.1%	0.031
Outpatient visit	4.3%	(−5.5,14.1)	**6.6%**	**(0.4,12.7)**	−2.3%	(−13.8,9.3)	6.5%	(−3.9,16.8)	**7.3%**	**(0.7,14.0)**	−0.9%	(−13.2,11.4)	90.5%	−1.0%	0.037
Emergency room visit	*−8.8%*	*(−17.8,0.2)*	**−9.0%**	**(−14.6,-3.5)**	0.3%	(−10.3,10.8)	*−9.3%*	*(− 19.0,0.4)*	**− 9.4%**	**(−15.6,-3.3)**	0.1%	(−11.3,11.6)	7.2%	2.1%	0.061
Hospital admission	**4.5%**	**(0.6,8.3)**	*2.5%*	*(−0.3,5.2)*	2.0%	(−2.7,6.7)	*2.8%*	*(−0.3,6.0)*	2.1%	(−0.8,5.0)	0.7%	(−3.5,5.0)	2.3%	32.1%	0.041
Care not sought b/c of cost	**−33.3%**	**(−65.7,-1.0)**	**−12.1%**	**(−23.8,-0.4)**	− 21.2%	(−55.6,13.2)	*−36.9%*	*(−74.9,1.2)*	*−12.3%*	*(− 25.8,1.2)*	− 24.6%	(− 62.3,13.2)	88.0%	− 27.9%	0.367
All care not received due to cost	**22.0%**	**(9.1,34.8)**	**15.9%**	**(6.8,24.9)**	6.1%	(−9.6,21.8)	**17.9%**	**(4.0,31.8)**	**13.0%**	**(3.1,22.9)**	4.9%	(−11.9,21.8)	30.0%	16.5%	0.078
**Adult Chronic Illness**															
Sought and received medical care	2.3%	(−7.7,12.3)	4.4%	(−3.8,12.6)	−2.1%	(−15.0,10.8)	6.4%	(−4.6,17.5)	2.7%	(−5.7,11.0)	3.8%	(−8.7,16.3)	93.9%	4.0%	0.042
Ever faced difficulties obtaining meds	0.0%	(−21.4,21.5)	11.7%	(−2.7,26.1)	−11.6%	(−37.5,14.2)	−10.6%	(−34.2,13.1)	14.2%	(−3.2,31.7)	*− 24.8%*	*(−53.6,4.0)*	73.6%	− 33.7%	0.072
General practitioner visit(s)	**−20.3%**	**(−39.8,-0.7)**	**−15.3%**	**(−30.2,-0.3)**	−5.0%	(− 29.6,19.6)	**− 23.1%**	**(−45.9,-0.3)**	***−27.6%***	***(− 42.7,-12.5)***	4.5%	(− 22.9,32.0)	62.5%	7.2%	0.096
Specialist visit(s)	−7.8%	(−30.0,14.4)	**−14.0%**	**(− 26.6,-1.5)**	6.3%	(−19.2,31.8)	− 14.4%	(− 38.3,9.5)	*− 13.6%*	*(− 27.8,0.5)*	−0.7%	(− 29.1,27.6)	70.2%	−1.1%	0.089
Hospital visit(s)	0.2%	(−16.0,16.4)	0.1%	(−13.1,13.3)	0.0%	(−20.8,20.9)	−1.5%	(− 22.2,19.2)	−9.3%	(− 24.4,5.7)	7.9%	(− 18.6,34.4)	27.5%	28.6%	0.118
Care not sought b/c of cost	100%	–	0.0%	–	100%	–	100.0%	–	0.0%	–	100%	–	82.6%	–	1.000
All care not received due to cost	6.0%	(−17.3,29.3)	***27.6%***	***(14.3,40.9)***	−21.6%	(−48.4,5.3)	−3.2%	(− 29.6,23.3)	**25.1%**	**(9.3,40.8)**	*−28.2%*	*(−59.1,2.6)*	40.2%	−70.3%	0.103
Cannot afford medication	2.6%	(−21.5,26.7)	**19.2%**	**(4.1,34.3)**	− 16.6%	(−45.1,11.8)	−5.1%	(−31.1,20.9)	**22.1%**	**(3.6,40.5)**	*−27.2%*	*(−59.5,5.0)*	62.0%	−43.9%	0.078

DiD (difference-in-difference) represents the difference in baseline to endline change between MPC and control groups; effect size indicates the magnitude of this difference (calculated by dividing the DiD estimate by overall mean)

Bold italic indicates statistically significant (*p* < 0.001) findings; bold indicates statistically significant (*p* < 0.05) findings; italic indicates statistically significant (*p* < 0.10) findings

^a^ Adjusted analyses controlled for principal applicant sex, age, education level, and marital status; household size and composition including the presence of household members in need of daily support, with chronic conditions, and children under five years old; dependency ratio; total household expenditure in the prior month; residence type; household crowding; and receipt of humanitarian assistance, specifically total value of cash assistance received in the prior month, and current WFP beneficiary status/transfer modality (e-voucher or unconditional cash)

^b^ DiD estimates are the modeled interaction effect of study group (i.e., MPC vs. control) and time period (i.e., baseline vs. endline)

With respect to child illnesses, households receiving MPCs reported children needing medical care more recently than control households (Fig. 1). Care-seeking rates for childhood illness were similarly high at baseline and endline for both groups, with more than 91% of households reporting having received care and no significant changes in either group. Among those that did not seek care, cost was the most commonly reported reason. Small sample sizes for those not seeking care precluded robust analysis of change over time; however, a significantly increasing proportion of both MPC and control households that sought care for a child’s illness reported not receiving all recommended care due to cost. In adjusted models comparing change in the two groups, the proportion of households not receiving all needed care due to cost increased by 19.3% (CI: 7.3,31.20%; *P* = 0.002) more in the MPC group compared to controls (effect size: 78.3%).

While most children received care as outpatients, at baseline 14% of MPC children and 16% of control children were treated at an emergency room and the remaining 3 and 1% of children, respectively, had a hospital admission. At endline, increases in hospitalizations were observed in both groups, however the MPC group saw a smaller increase as compared to controls (adjusted difference − 6.1%, CI: − 11.7,-0.4%; *P* = 0.037; effect size: − 133.5%). While emergency room visits significantly decreased in both groups in adjusted analyses, the difference in change between MPC and control households was not statistically significant (*P* = 0.730). Access to medication for childhood illness was also very high, though no statistically significant changes were observed within or between groups.

For adults with acute illness, households receiving MPCs reported needing care more recently compared to controls (Fig. 1); these differences were significant only at baseline. Care-seeking rates were similarly high between MPC and control households at baseline (88.3 and 90.6%, respectively) and increased among MPCs but decreased among controls for a marginally significant difference in change of 11.3% (CI: − 0.3,22.9%; *P* = 0.057; effect size: 12.7%). Cost was consistently the primary reason for not seeking care, and also an increasingly important reason for not receiving all recommended care. While 19% of households in both groups reported care averted due to cost at baseline, this proportion increased to 41.1% among MPC households and 35.2% of controls at endline, though the difference in change was not statistically significant (*P* = 0.566). Care-seeking location for acute illness was consistently similar between the two groups but changed over time, albeit not significantly, with an increase in outpatient care-seeking and hospitalizations and a decrease in emergency room visits. Access to medication for adult acute illness was very high, with more than 97% of households in both groups reporting they were able to obtain prescribed medications and no statistically significant change within each group nor difference between groups.

The proportion of households with member(s) with chronic health conditions was similar at baseline and endline between MPC and control households. Among household members with chronic condition(s) the most frequently reported were hypertension, diabetes, and cardiovascular disease. Households receiving MPCs reported seeking care for adult chronic illnesses more recently compared to controls (Fig. 1), though this difference was significant only at endline. Similarly, high care-seeking rates were observed in both study groups at baseline and endline; more than 90% of individuals with a chronic condition received medical care in Lebanon for their condition. Individuals with chronic illnesses in MPC households were more able to afford all recommended care as compared to those in control households. The proportion of households not receiving all recommended care due to cost increased among both MPC recipients and controls during the study period; however, in adjusted analyses, this proportion decreased among MPC households (− 3.2%, CI: − 29.6,23.3%; *P* = 0.814) and increased significantly among controls (25.1%, CI: 9.3,40.8%; *P* = 0.002). Accordingly, the adjusted difference in change in the proportion of MPC households not receiving recommended care due to cost compared to controls was − 28.2% (CI: − 59.1,-2.6%; *P* = 0.073; effect size: − 70.3%). Visits to a general practitioner, specialist physician, and hospital all decreased from baseline to endline in both groups and while the decrease in visits both to a GP and to a specialist were statistically significant among controls, there were no significant differences in change between groups.

Access to medication for adult chronic illness was differentially affected by receipt of MPCs with adjusted analyses showing marginally significant benefits for MPC households relative to controls. The proportion of households facing difficulties obtaining medication for chronic illness decreased in MPC households (adjusted change of − 10.6%, CI: − 34.2,13.1$; *P* = 0.379) but increased among controls (adjusted change of 14.2%, CI: − 3.2,31.7%; *P* = 0.109) with a marginally significant difference in change of − 24.8% (CI: − 53.6,4.0%; *P* = 0.091) for MPC recipients compared to controls (effect size: − 33.7%). Similarly, MPC households unable to afford chronic disease medication decreased, though not significantly, over follow-up (adjusted change of − 5.1%, CI: − 31.1,20.9%; *P* = 0.814) and increased significantly among controls (adjusted change of 22.1%, CI: 3.6,40.5%; *P* = 0.019); the adjusted difference in change between MPC and control households was − 27.2% (CI: − 59.5,5.0%; *P* = 0.097; effect size: − 43.9%).

### Health expenditures

**Fig. 3:**
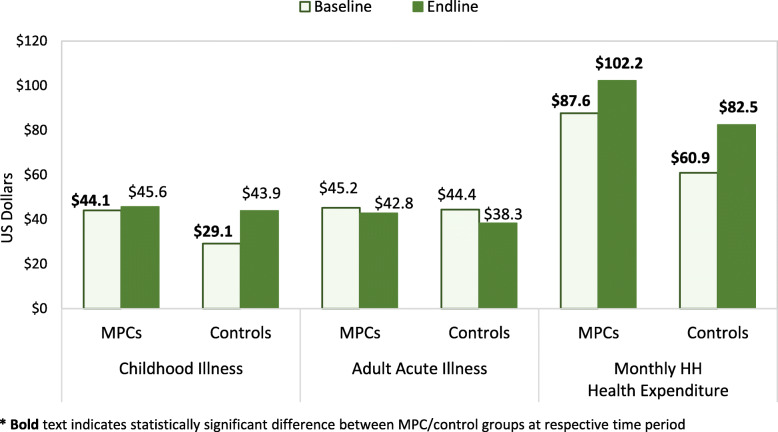
Health Expenditures by Group (one-year period from baseline to endline)

Health expenditures were also evaluated for the most recent household member illness (within the past 6 months) for children and both acute and chronic illness among adults; overall household health expenditures were also assessed. Baseline and endline values for each group are provided in Additional file 4; individual change over time and differences in change between study groups are presented in Table 5; baseline and endline mean overall costs for child and adult acute illness, as well as overall household health expenditures are presented in Fig. 3.

**Table 5:** Change in log health expenditures for most recent care and in the preceding month ^a^

	UNADJUSTED CHANGE OVER TIME						ADJUSTED CHANGE OVER TIME ^b^								
	MPC HHs		Control HHs		DiD		MPC HHs		Control HHs		DiD ^**c**^		Overall Mean/%	Effect Size	R^**2**^
	%	(95% CI)	%	(95% CI)	%	(95% CI)	%	(95% CI)	%	(95% CI)	%	(95% CI)			
**Most Recent Childhood Illness**															
**Health Facility Payments for OP Care**^**d**^															
Any payment for OP care at facility	3.1%	(−5.6,11.8)	0.6%	(− 5.1,6.3)	2.5%	(−7.9,12.9)	4.3%	(−5.1,13.7)	−0.2%	(−6.3,5.9)	4.5%	(−6.7,15.8)	87.8%	5.2%	0.027
Total paid at facility for visit (all HHs)	0.19	(−0.9,1.3)	0.20	(−0.5,0.9)	−0.01	(−1.3,1.2)	0.34	(−0.8,1.5)	− 0.02	(− 0.7,0.7)	0.36	(− 1.0,1.7)	1.37	26.2%	0.035
**Medication Costs at Pharmacy/Elsewhere**															
Any payment for medication outside facility	**15.7%**	**(4.4,27.0)**	**15.0%**	**(6.2,23.8)**	0.7%	(−13.6,15.0)	**14.8%**	**(2.2,27.4)**	4.8%	(−5.0,14.7)	9.9%	(−6.0,25.8)	57.0%	17.4%	0.059
Total paid for medication (all HHs)	**1.61**	**(0.2,3.0)**	**1.81**	**(0.7,2.9)**	−0.19	(−1.9,1.6)	**1.54**	**(0.0,3.1)**	0.51	(−0.7,1.7)	1.03	(−0.9,3.0)	−2.49	−41.3%	0.058
**Total Amount Paid for Illness**^**e**^															
Any expense for most recent illness	4.6%	(−4.0,13.3)	**6.7%**	**(0.5,12.9)**	−2.0%	(−12.7,8.6)	5.5%	(−3.9,15.0)	5.1%	(−1.9,12.2)	0.4%	(−11.0,11.8)	86.3%	0.5%	0.048
Total cost for most recent illness (all HHs)	0.59	(−0.5,1.7)	**1.01**	**(0.2,1.8)**	−0.41	(−1.8,0.9)	0.71	(−0.5,1.9)	0.64	(−0.3,1.5)	0.07	(−1.4,1.5)	1.52	4.6%	0.056
**Most Recent Acute Adult Illness**															
**Health Facility Payments for OP Care**^**d**^															
Any payment for OP care at facility	**−11.9%**	**(−20.8,-2.9)**	0.7%	(−6.5,7.9)	**−12.6%**	**(−24.1,-1.1)**	**− 14.5%**	**(− 23.4,-5.5)**	−1.1%	(−9.0,6.8)	**− 13.4%**	**(− 25.4,-1.3)**	88.1%	−15.2%	0.055
Total paid at facility for visit (all HHs)	**−1.34**	**(− 2.4,-0.3)**	−0.01	(− 0.8,0.8)	**− 1.34**	**(−2.7,0.0)**	**− 1.57**	**(− 2.7,-0.5)**	− 0.33	(−1.3,0.6)	*− 1.24*	*(−2.7,0.2)*	1.64	−75.6%	0.057
**Medication Costs at Pharmacy/Elsewhere**															
Any payment for medication outside facility	−6.6%	(−21.1,7.9)	1.9%	(−8.5,12.4)	−8.6%	(−26.4,9.3)	− 11.3%	(− 26.7,4.2)	−3.5%	(− 15.2,8.3)	− 7.8%	(− 27.0,11.5)	56.6%	− 13.7%	0.044
Total paid for medication (all HHs)	−0.92	(− 2.8,0.9)	− 0.04	(− 1.3,1.3)	− 0.88	(− 3.1,1.4)	− 1.37	(−3.3,0.6)	− 0.67	(− 2.1,0.8)	−0.70	(− 3.1,1.7)	− 2.45	28.6%	0.047
**Total Amount Paid for Illness**^**e**^															
Any expense for most recent illness	**−12.1%**	**(−22.8,-1.5)**	−0.6%	(− 8.8,7.5)	*− 11.5%*	*(− 24.9,1.9)*	**− 13.2%**	**(− 24.2,− 2.1)**	-2.1%	(− 11.5,7.3)	− 11.1%	(− 25.5,3.3)	84.9%	− 13.1%	0.046
Total cost for most recent illness (all HHs)	**−1.77**	**(− 3.2,-0.4)**	− 0.05	(− 1.1,1.0)	*−1.71*	*(− 3.5,0.0)*	**−1.95**	**(− 3.4,-0.5)**	− 0.28	(−1.5,0.9)	*− 1.67*	*(− 3.5,0.2)*	1.49	− 111.7%	0.059
**Most Recent Adult Chronic Illness Visit**															
**Health Facility Payments for OP Care**^**d**^															
Any payment for OP care at facility	5.0%	(−10.8,20.9)	*10.6%*	*(−1.9,23.1)*	−5.6%	(− 25.8,14.6)	− 1.5%	(− 19.2,16.3)	7.2%	(− 8.2,22.6)	− 8.7%	(− 33.2,15.9)	77.5%	−11.2%	0.073
Total paid at facility for visit (all HHs)	0.57	(−1.4,2.6)	1.32	(− 0.3,3.0)	− 0.74	(−3.3,1.9)	− 0.10	(− 2.4,2.2)	0.69	(− 1.2,2.5)	− 0.78	(− 3.8,2.3)	0.51	− 154.2%	0.084
**Average Monthly Medication Costs**															
Any regular (monthly) medication costs	4.9%	(− 17.2,27.1)	5.1%	(−7.5,17.8)	−0.2%	(− 25.7,25.3)	5.2%	(− 19.1,29.4)	− 0.8%	(− 15.0,13.3)	6.0%	(−22.3,34.3)	71.6%	8.4%	0.077
Average monthly medication costs (all cases)	0.43	(−2.5,3.4)	0.61	(−1.0,2.2)	−0.18	(−3.5,3.2)	0.32	(−2.9,3.5)	−0.02	(−1.8,1.8)	0.33	(−3.4,4.0)	−0.07	− 449.8%	0.086
**Routine Spending on Health**															
Health expenditures (past month)^f^	0.70	(−0.2,1.6)	***1.49***	***(0.8,2.2)***	−0.79	(−1.9,0.3)	0.24	(−0.7,1.2)	0.53	(−0.2,1.3)	−0.30	(−1.5,0.9)	2.01	−14.7%	0.131
Sold assets to pay for health (past 3 mos)	2.5%	(−1.5,6.5)	***7.0%***	***(3.6,10.3)***	*−4.5%*	*(−9.7,0.7)*	2.0%	(−2.4,6.4)	**4.0%**	**(0.4,7.7)**	−2.0%	(−7.9,3.8)	6.8%	−29.7%	0.044
Borrowed to pay for health (past 3 mos)	***42.4%***	***(32.8,52.0)***	***34.9%***	***(29.0,40.8)***	7.5%	(−3.8,18.7)	***38.0%***	***(27.7,48.3)***	***32.6%***	***(25.8,39.4)***	5.4%	(−6.6,17.3)	37.3%	14.4%	0.218

DiD = difference-in-difference; HH = household; OP = outpatient

DiD represents the difference in baseline to endline change between MPC and control groups; effect size indicates the magnitude of this difference (calculated by dividing the DiD estimate by overall mean)

Bold italic indicates statistically significant (*p* < 0.001) findings; bold indicates statistically significant (*p* < 0.05) findings; italic indicates statistically significant (*p* < 0.10) findings

^a^ Analyzed as log US$; exchange rate: 1507.5 LBP = 1 US$

^b^ adjusted analyses controlled for principal applicant sex, age, education level, and marital status; household size and composition including the presence of household members in need of daily support, with chronic conditions, and children under five years old; dependency ratio; total household expenditure in the prior month; residence type; household crowding; and receipt of humanitarian assistance, specifically total value of cash assistance received in the prior month, and current WFP beneficiary status/transfer modality (e-voucher or unconditional cash)

^c^ DiD estimates are the modeled interaction effect of study group (i.e., MPC vs. control) and time period (i.e., baseline vs. endline)

^**d**^ includes consultation fees, diagnostic testing and medications obtained at health facility during the initial visit to health facility, hospital outpatient department, or emergency room (without overnight stay)

^e^ includes health facility payments for outpatient care and medications purchased at pharmacies outside health facilities (does not include referrals, in-patient care, and transportation)

^f^ at facilities and for medication

The proportion of households incurring out-of-pocket costs for the most recent child illness increased among MPC recipients with change not significantly differing from controls in all cost categories. While adjusted change in households with facility payments or incurring any payments (i.e., to health facilities or for medication) did not significantly change in either group, the proportion of households incurring medication costs increased significantly among MPC recipients (adjusted change 14.8%, CI: 2.2,27.4%; *P* = 0.022) but not significantly for controls (4.8%, CI: − 5.0,14.7%; *P* = 0.337); this change did not significantly differ between groups (*P* = 0.221). Adjusted differences between groups’ changes in payment amounts were not statistically significant for any expenditure category (facility payment *P* = 0.595, medication payment *P* = 0.299, overall payment *P* = 0.925).

The adjusted proportion of households incurring payments at facilities for adult acute illness outpatient care decreased significantly among MPC households (− 14.5%, CI: − 23.4,-5.5%; *P* = 0.002) and nominally decreased among controls (− 1.1%, CI: − 9.0,6.8%; *P* = 0.785), with an adjusted DiD of − 13.4% (CI: − 25.4,-1.3%; *P* = 0.030; effect size: − 15.2%). Overall payments also decreased significantly among MPC recipients (− 13.2%, CI: − 24.2,-2.1%; *P* = 0.020) but neither change among controls (− 2.1%, CI: − 11.5,7.3%; *P* = 0.666) nor the difference in change between groups were significant (*P* = 0.131). Accordingly, change in facility payments among MPC recipients were only 0.29 (CI: − 0.12,0.70; *P* = 0.088) times that of controls and change in total expenditures for recent care among MPCs was 0.19 (CI: − 0.16,0.54; *P* = 0.078) times the change among controls. No significant differences between MPC and control households were observed in the amount (*P* = 0.428) nor in the proportion (*P* = 0.569) of households with medication payments for adult acute illnesses.

Costs associated with adult chronic illness were less common than those for child and adult acute illnesses, but similar between study groups. The proportion of households with out-of-pocket payments for the most recent visit for chronic illness care increased from baseline to endline in both groups; however, neither change within each group (MPC adjusted change *P* = 0.871; control adjusted change *P* = 0.357) nor the difference in change between groups (adjusted DiD *P* = 0.487) were statistically significant. Out-of-pocket payment amounts for care increased for control participants but decreased among MPC recipients, though again no significant change was observed in either group (MPC *P* = 0.934; control *P* = 0.462) or in the adjusted DiD (*P* = 0.614). Conversely, the proportion of participants paying for monthly chronic illness medication increased in MPC recipients and decreased among controls during the study period, but not significantly (adjusted MPC *P* = 0.675; control *P* = 0.908; DiD *P* = 0.677). Monthly medication payments also increased for MPC recipients, yet decreased for controls, however, adjusted group change (MPC *P* = 0.846; control *P* = 0.984) and difference in change between study groups (*P* = 0.858) were again not statistically significant.

Total household health expenditures in the past month were significantly higher among MPC households in both periods (Fig. 3) and increased, although not significantly, from baseline to endline among both MPC and control households. Neither adjusted change in routine health spending within each group nor the between-groups difference in change were statistically significant (adjusted MPC change *P* = 0.634, controls change *P* = 0.175, DiD *P* = 0.638).

Relatively few households reported selling assets to pay for health expenses in the past 3 months in either group. Although asset sales increased among both MPC recipients and controls during the study period, change was only statistically significant in controls (MPC adjusted *P* = 0.373; control adjusted *P* = 0.030) and no significant difference in change between groups was observed (adjusted *P* = 0.496). Borrowing money to pay for health expenses was far more common in both groups, with significantly more MPC households borrowing money at both time periods compared to controls. Borrowing increased significantly from baseline to endline both among MPC households (38.0%, CI: 27.7,48.3%; *P* < 0.001) and controls (32.6%, CI: 25.8,39.4%; *P* < 0.001) in adjusted analysis; however, the adjusted difference in borrowing changes was not statistically significant between groups (*P* = 0.376).

## Discussion

### Careseeking

While baseline levels of care-seeking for child illness and acute illnesses among adults were high, MPC households reported needing care more frequently for child illness and both adult acute and chronic illnesses at baseline and endline; however, adjusted differences in change in care-seeking rates over time were not statistically significant (child illness) and marginally significant (adult acute illness), suggesting that receipt of MPCs may increase care-seeking for health services only initially. Across all categories of care, cost was the primary barrier among those that did not seek care and among care recipients for both MPC and control households; many reported not receiving all recommended care due to cost. Over the year-long study period, increases in the proportion of households not receiving all needed care were observed for both MPCs and controls for child illness (significant, *p* < 0.05) and adult acute and chronic illness (marginally significant, *p* < 0.10). The increase in care averted due to cost in both groups for child and adult acute illness suggests that access to services outside the basic package of subsidized care is limited and potentially deteriorating. This observation aligns with UNHCR’s annual Health Access and Utilization Survey (HAUS), which showed consistent increases in household health expenditures over time, starting at US$90 in 2014 and increasing annually to reach an average of US$157 in 2019; however, declines in health access observed in the present study are contrary to increases in care access for acute and chronic illnesses demonstrated in the 2019 HAUS and overall improvements reported in the 2019 Vulnerability Assessment of Syrian Refugees in Lebanon (VASyR) [20, 22]. One possibility is that the observed increase in monthly health expenditures is the result of changes in cost sharing policies, where increased out of pocket payments present a financial barrier to care.

### Medications

MPCs did not enhance household ability to obtain all needed medications for child and adult acute illness, but they may be beneficial for adult health where adjusted differences in change from baseline to endline for MPC recipients compared to controls of − 24.8% for difficulty obtaining needed medications for chronic illness and − 27.2% for inability to afford chronic illness medications were observed (*P* < 0.10 for all comparisons). These findings suggest MPCs may improve access to medication for adult chronic illness; one potential explanation for this is that costs associated with chronic health conditions are incurred more routinely, thus households receiving MPCs may be better able to budget and plan for these expenses. Evidence on the impact of MPCs and health access is limited, however our findings contradict a 2020 impact evaluation of MPC assistance in Lebanon, which found significant benefits of long-term MPC on child health access [23]. Conversely, our findings are consistent with other regional evidence, where evaluations revealed inconsistent or insignificant impacts of MPC on health access. Notably, a 2019 analysis of the Emergency Social Safety Net (ESSN) Program for Syrian refugees in Turkey found no statistically significant differences in care-seeking by cash beneficiary status [24]. Similarly, two evaluations of UNHCR/UNICEF cash assistance for Syrian refugees in Jordan reported no strong association between receipt of cash assistance and both care-seeking and access to health services [25, 26].

### Health expenditures

With regard to health expenditures, the proportion of MPC recipients that reported out of pocket expenses for both the most recent child and adult acute illness increased significantly over the study period as did the [log transformed] total visit cost for the most recent illness. In contrast, log health expenditures in control group households did not change for child and adult acute illness. At endline, total expenditures for the most recent child and adult acute illness were similar in both controls and MPC households averaging US$38–46 per visit, which is well beyond costs expected for subsidized services in the essential care package [5]. Both MPC recipients and controls reported sizable increases in monthly household spending on health from baseline to endline (17 and 15%, respectively) with mean monthly health expenditures of US$102 and US$83 at endline, respectively; difference in change between the two groups was not significant, likely due to control households having lower baseline expenditures. While control households had lower monthly health expenditures at endline, the comparable increase in spending over the year study period indicates they were able to find the means to increase health expenditures despite limited budgets and other competing priorities. Both MPC and control households reported significant increases in borrowing to pay for health expenses over the study period (38 and 33%, respectively) and there was no significant difference in magnitude of change for borrowing or asset sales between the two groups, suggesting that MPC was not protective for household financial risks associated with health. The parallel MPC study in Jordan also reached a similar conclusion [24].

In the context of previous literature, overall health expenditures by Syrian refugee households remained relatively stable from 2017 through 2019 according to the 2019 VASyR but consistently increased per the 2019 HAUS [20, 22]. Moreover, HAUS reports of expenditure for chronic and acute illness from 2017 to 2019 similarly varied, suggesting inconsistent findings in our study are not entirely out of place with overall trends over time and mirror the absence of significant impact of MPCs on health expenditures observed in a 2020 impact evaluation of MPCs in Lebanon [23]. Mixed results were also reported in the 2018 evaluation of Lebanon’s Min Ila cash program for children where expenditures on health for young children (5–9 years) were comparably higher among beneficiary households relative to controls, but differences in health expenditures for children 10–17 years were not significant [27]. Both small transfer size and variable health needs are potential explanations for the mixed findings on health outcomes associated with MPC in Lebanon. Transfer values may be insufficient to realize gains in health among vulnerable households who may prioritize other essential expenditures, such as food and housing, which in many instances are more predictable than health care expenses. In this study, total monthly cash assistance in the MPC group averaged approximately US$300 per household, which equates to approximately 38% of average monthly expenditures. However, when considering the minimum expenditure basket of US$104 per person per month [28]^5^ and average size of households in the MPC group (7.5 members), it is apparent that expenditures in many households do not cross this threshold. Thus, transfer size, albeit large relative to overall expenditures, may be insufficient to see significant gains in health indicators because poverty persists despite ongoing receipt of humanitarian assistance.

### Hospitalizations

MPC receipt was associated with consistent hospitalization rates for child illness compared to an increasing hospitalization among controls, translating to a significant difference in change in households with a child hospitalization (− 6.1%) among MPC recipients compared to controls; no significant differences in change in emergency room visits or hospitalizations were observed for adult acute or chronic illness. These observations are in contrast to another recent MPC study in Lebanon which found that long-term, but not short-term recipients of cash were more likely to need hospitalization than controls (which also could be result of targeting practices that favor households with chronic medical conditions and medically vulnerable individuals) [23]. Significant decreases in emergency room utilization for the most recent child and adult acute illness in both the MPC and control groups is likely attributable to July 2018 changes in UNHCR policies for out of pocket payments for hospitalization, where patient shares for lower-cost hospital care increased, thereby incentivizing care-seeking at primary level facilities for less urgent cases or, potentially, disincentivizing seeking hospital care [5]. In the parallel UNHCR MPC study in Jordan, significant differences in change in hospital admissions for both adult acute and chronic illnesses were observed between MPC and non-MPC recipients, indicating that MPC receipt may reduce hospitalization, albeit among different groups, in multiple settings . This is potentially important from a policy and cost perspective, where unrestricted cash transfers have the potential to reduce hospitalization costs, which exceeded US$53 million for refugees in Lebanon in 2017 [29].

### Humanitarian health funding

In 2019, a total of US$144 million was allocated through the Lebanon Crisis Response Plan to support the overall health sector response in Lebanon [30]. In the present study, monthly household expenditures among MPC recipient households averaged US$19.7 (endline) to US$26.7 (baseline) more than control households, which translates to a difference in household health expenditures of US$236.4 to US$320.4 annually. Applying these figures to the 55,000 Syrian refugee households in Lebanon that receive MPC [23], it can be estimated that MPC expenditures contribute US$13.0–17.6 million annually to refugee health in Lebanon, equating to approximately 10% of health sector response funding. While MPC increased spending on health they cannot be viewed as a replacement to direct support to the health sector, though they are one of several mechanisms to remove financial access barriers. There are few comparative studies of MPC compared to conditional cash transfers, which either have qualifying criteria (e.g. for individuals with chronic health conditions) or use conditions (e.g. ability to demonstrate care-seeking or medication purchase); however, evidence from Syrian refugees in Jordan suggests that conditional cash transfers and health education may be more effective in improving health indicators for chronic disease compared to MPC [31].

### Limitations

While efforts were made to design and implement a rigorous study, as with all research, findings must be interpreted in the context of certain limitations. Recalibration of UNHCR targeting criteria mid-way through the study resulted in changes in intervention receipt for many study households, which substantially decreased the final MPC recipient sample size. This led to low precision of some estimates and reduced the ability to detect statistically significant differences for many outcomes due to relatively small sample sizes in addition to prohibiting statistical comparisons of reasons for not seeking care. Moreover, it cannot be assumed that there were no systematic differences between refugees receiving, or not receiving, MPC the entire study period and those transitioning onto or off of MPC during the study. Additionally, changes to UNHCR’s referral care program, specifically changes to the cost-sharing ratio between patient-share and UNHCR-share, immediately following baseline data collection may have influenced hospital utilization and associated costs for all refugees independent of MPC beneficiary status, in addition to adding difficulty to interpreting results. With respect to data quality, respondent-reported expenditures pose potential limitations for several reasons. While enumerators were provided thorough training on probing for reliable responses and interviews were supervised throughout data collection, the potential for recall bias and/or respondents misunderstanding what should be included in questions about various expenditures remain. Finally, it would have been ideal to conduct the study when cash transfers were being scaled up such that the MPC group was comprised of new beneficiaries, however, given the longstanding nature of the crisis in Syria and the need to conduct research within the context of an ongoing humanitarian response, this was not possible.

## Conclusions

This study assessed care-seeking, medication access, and health expenditures among MPC recipients and similar control households and observed relatively few statistically significant differences in outcomes using adjusted models to compare change over time between the two groups. MPCs did not appear to be protective with respect to use of financial coping mechanisms to pay for health expenses including borrowing and asset sales despite the relatively large transfer size, which was equivalent to 38% of monthly household expenditures on average. MPC recipients reported higher monthly household health expenditures, however, transfer values may not have been sufficiently large to increase access to more costly services or reduce the household economic impacts of larger health expenses given increasingly high poverty levels. Increases in care averted due to cost in both groups for child and adult acute illness, combined with increasing impoverishment, suggests potentially deteriorating access to services outside the subsidized care package, and shows the situation’s limited sustainability. Among adults with chronic health conditions, MPCs were marginally associated with an increase in ability to afford all recommended services and fewer difficulties in obtaining and affording chronic disease medication, which is an important finding that demonstrates that unrestricted cash transfers may be sufficient for improving access to care and medications for chronic illness. While there is little evidence available to date on this topic, the parallel UNHCR MPC study conducted in Jordan did not observe any benefits regarding care averted due to cost or accessibility and affordability of medications for chronic diseases, suggesting that MPC outcomes may be context-specific and influenced by a myriad of factors . While hospitalizations for child illness were averted by MPC receipt in Lebanon, a similar pattern was noted for hospitalizations for adult acute and chronic illness in Jordan, suggesting that further analysis of unaffordable hospitalization costs could be useful for informing resource allocation. Findings from this study among Syrian refugees in Lebanon, while mixed, suggest unconditional cash transfers may improve access to health services and medication for chronic diseases, in addition to reducing hospitalizations among children, but overall may be insufficient on their own to address health utilization and expenditures. MPC may be a good strategy to support health when implemented when sufficient transfer values are realized in conjunction with additional targeted conditional cash transfers for health and other health sector interventions such as health education and subsidized care costs.

## Supplementary Information

**Additional file 1: Supplemental Methods.** Description: Additional details on sampling methods including economic variability of severely vulnerable households by intervention and the sub-set of households that served as the reference population for the sample, change in intervention receipt among study households during the study period, and analyzed sample follow-up by intervention receipt.

**Additional file 2.** Multi-purpose cash transfers and health among vulnerable Syrian refugees in Lebanon: Study Questionnaire. Description: The questionnaire developed and used for this study.

**Additional file 3.** Health Care-Seeking and Medicines for Household Member Illness at Baseline and Endline. Description: Baseline and endline descriptive analyses of care-seeking outcomes by group.

**Additional file 4.** Health Expenditures for Most Recent Child, Adult Acute, and Adult Chronic Illness Care and in the Preceding Month (USD) at Baseline and Endline. Description: Baseline and endline descriptive analyses of health expenditure outcomes by group.

## Data Availability

The datasets supporting the conclusions of this article are available in the Humanitarian Data Exchange, and [upon acceptance] can be accessed at https://data.humdata.org/dataset/mpc-for-syrian-refugee-health-in-lebanon.

## Abbreviations

CVA: Cash and voucher assistance
DiD: Difference-in-difference
ESSN: Emergency Social Safety Net
HAUS: Health Access and Utilization Survey
JHSPH: Johns Hopkins Bloomberg School of Public Health
LBP: Lebanese pounds
MPCs: Multipurpose cash transfers
UNHCR: United Nations High Commissioner for Refugees
UNICEF: United Nations International Children’s Fund
US$: U.S. Dollars
VASyR: Vulnerability Assessment of Syrian Refugees in Lebanon
WFP: World Food Program

## Footnotes

1 Households in each of these groups are targeted for assistance based on vulnerability classification determined by proxy means testing or a ‘desk formula’ taking into consideration a range of characteristics obtained during registration.

2 All households in both groups received WFP e-cards for food assistance, valued at $27/person/month

3 50% was identified because this proportion provided significant differences of the same magnitude from any other proportion that could be detected, thus ensuring the ability to detect significant differences for multiple indicators.

4 As the study period ended prior to the compounded economic and financial crisis (beginning October 2019), COVID-19, and the August 2020 explosion at the Port of Beirut, findings do not reflect the substantial impact of these events on the context.

5 The minimum expenditure basket reflects the per capita monthly amount needed to meet basic needs.
