# Supplementary material for: Multi-purpose cash transfers and health among vulnerable Syrian refugees in Lebanon: a prospective cohort study
Source: BMC Public Health. 2021 Jun 19;21:1176. doi: 10.1186/s12889-021-11196-8 (PMC8214292; doi:10.1186/s12889-021-11196-8)
Supplement: Supplementary file 1 — Additional file 1: Supplemental Methods. Description: Additional details on sampling methods including economic variability of severely vulnerable households by intervention and the sub-set of households that served as the reference population for the sample, change in intervention receipt among study households during the study period, and analyzed sample follow-up by intervention receipt. [file 12889_2021_11196_MOESM1_ESM.pdf]

Supplemental Methods

Sampling. Eligibility for cash transfers is based on a households' vulnerability status, which is determined by comparing projected household expenditures to the minimum expenditure basket (MEB) reflecting the needs and average expenditures for a Syrian household. Household characteristics collected during registration and subsequent updates are used in conjunction with a desk formula, developed based on data from the Vulnerability Assessment of Syrian Refugees (VASyR), to estimate average monthly per capita expenditures. Households with per capita expenditures estimated below the MEB* are classified as vulnerable or severely vulnerable and are eligible to receive multipurpose cash transfers, food assistance, and other benefits such as reduced cost health services. The economic variability of severely vulnerable households by intervention and the sub-set of households that served as the reference population for the sample are presented in Table 1. Change in receipt of MPC among study households during the study period is presented in Table 2. Follow-up details, including reasons for loss to follow-up, are provided in Table 3.

Table 1: Registered Syrian Refugee Households Eligible for MPCs and Study Inclusion

	Bekaa	BML	North	South
Severely Vulnerable Households Eligible for MPC				
UNHCR MPC Recipients (%)	18,975 (58.0%)	4,090 (12.5%)	7,534 (23.0%)	2,097 (6.4%)
Expenditure Mean (median)	49.3 (49.0)	66.7 (68.2)	61.0 (62.1)	56.9 (60.6)
Expenditure Range	25.0-73.4	41.0-74.5	32.3-74.1	24.5-71.2
Non-MPC Recipients (%)	29,582 (42.6%)	12,098 (17.4%)	21,536 (31.0%)	6,201 (8.9%)
Expenditure Mean (median)	69.6 (71.3)	80.3 (81.1)	76.0 (76.2)	77.6 (79.5)
Expenditure Range	26.3-87.0	41.8-87.0	39.0-87.0	21.4-81.0
Households Eligible for Inclusion in the Sample (estimated expenditure of US$60.00-69.99)				
UNHCR MPC Recipients (%)	1,710 (17.5%)	2,108 (21.6%)	4,952 (50.7%)	1,000 (10.2%)
Expenditure Mean (median)	64.6 (65.8)	66.0 (66.4)	64.0 (64.0)	65.7 (65.9)
Expenditure Range	60.0-69.5	60.0-70.0	60.0-69.9	60.0-70.0
Non-MPC Recipients (%)	7,037 (64.6%)	294 (2.7%)	3,273 (30.0%)	297 (2.7%)
Expenditure Mean (median)	66.3 (67.3)	65.9 (66.5)	67.7 (68.1)	66.8 (68.0)
Expenditure Range	60.0-70.0	60.1-70.0	60.0-70.0	60.1-70.0

BML = Beirut/Mount Lebanon

* The minimum expenditure basket (MEB) "covers the basic needs of a household to live with dignity", whereas the survival minimum expenditure basket (SMEB) "covers the requirements to exist and meet lifesaving needs while displaced." The MEB and the SMEB calculations are based on the needs of a family of five and are determined on both a household and per capita basis. In Lebanon the per capita MEB and SMEB were calculated at US$110 and US$173, respectively, in 2016 (the most recent update at the time of study initiation).

Table 2: Change in Intervention Receipt among Study Households During the Study Period

	**Continue on Intervention**		**Continue as Control**		**Switch: Intervention to Control***		**Switch: Control to Intervention**	
	N	%	N	%	N	%	N	%
North	49	45.8%	84	22.4%	54	15.2%	18	29.5%
BML	15	14.0%	26	6.9%	48	13.5%	8	13.1%
Bekaa	32	29.9%	245	65.3%	225	63.4%	24	39.3%
South	11	10.3%	20	5.3%	28	7.9%	11	18.0%
Total	**107**	**11.9%**	**375**	**41.8%**	**355**	**39.5%**	**61**	**6.8%**

BML = Beirut/Mount Lebanon. * Excluded from final analysis

Table 3: Analyzed Sample Follow-Up by Intervention Receipt

	**Continue on Intervention** [a]		**Switch: Control to Intervention** [a]		**Continue as Control**		**Total Analyzed Sample**	
	N	%	N	%	N	%	N	%
Complete follow-up	107	98.2%	61	95.3%	375	84.5%	543	88.0%
Lost to follow-up	2	1.8%	3	4.7%	69	15.5%	74	12.0%
Refused endline interview	0	0.0%	0	0.0%	1	0.2%	1	0.2%
Ineligible	0	0.0%	1	1.6%	13	2.9%	14	2.3%
Unreachable	2	1.8%	2	3.1%	55	12.4%	59	9.6%
Total	**109**	**17.7%**	**64**	**10.4%**	**444**	**72.0%**	**617**	**100%**

[a] Included as MPC group for final analysis
