# Supplementary material for: Multi-purpose cash transfers and health among vulnerable Syrian refugees in Lebanon: a prospective cohort study
Source: BMC Public Health. 2021 Jun 19;21:1176. doi: 10.1186/s12889-021-11196-8 (PMC8214292; doi:10.1186/s12889-021-11196-8)
Supplement: Supplementary file 2 — Additional file 2. Multi-purpose cash transfers and health among vulnerable Syrian refugees in Lebanon: Study Questionnaire. Description: The questionnaire developed and used for this study. [file 12889_2021_11196_MOESM2_ESM.pdf]

Multi-purpose cash transfers and health among vulnerable Syrian refugees in Lebanon: Study Questionnaire

1. SECTION 1. INTERVIEW BACKGROUND INFORMATION
Date of Enrollment

2. Interviewer number

3. UNHCR Case Number from sampling list

4. Assigned sample group
Choose one response
- MPC
- Control

5. Household Study ID

6. Arabic consent script
7. Arabic consent script, continued

8. Do you agree to participate in this study?
Choose one response
- Yes, agree to participate
- No, refuse to participate   If this response, jump to 308
- Yes, agree to participate but asked to postpone interview   If this response, jump to 308

9. SECTION 2: INFORMATION ABOUT RESPONDENT AND UNHCR CASE
Identify the Principal Applicant of the case number; if not present, an adult included in the same registration case that is considered capable and reliable for the interview may be interviewed.   If possible, select the mother of the children or an adult who is engaged in decision making for the household.

10. Please tell me your UNHCR case number.
INTERVIEWER: Does the case number provided match the sampled case number?
If not, probe to find someone on the sampled case number. If someone on the sampled case number cannot be identified, select "no matching case number not identified" and end the interview.
Choose one response
- Yes, matching case number provided
- No, matching case number not identified   If this response, jump to 308

11. Record UNHCR case number

12. Given Name of Participant

13. Family Surname(s)

14. Cell phone number 1

15. Relationship to phone owner
Choose one response
- Shared household phone
- Self/personal phone

- Another household member's personal phone
- Neighbor
- Other

16. Alternate cell phone number where participant can be reached

17. Relationship to alternate phone owner

Choose one response
- Shared household phone
- Self/personal phone
- Another household member's personal
- Neighbor
- Other

18. Respondent sex

Choose one response
- Male
- Female

19. How old are you? (in years)

20. What is the highest level of education you have completed?

Choose one response
- No formal schooling
- Some primary school
- Primary school completed
- Preparatory school completed
- Secondary school completed
- College/ University completed
- Don’t Know
- Refused

21. What is your marital status?

Choose one response
- Married
- Widowed
- Never married
- Divorced
- Refused

22. What is your relationship to the principal applicant on the UNHCR case?

Choose one response
- I am the principal applicant   If this response, jump to 26
- Wife/Husband
- Mother/Father
- Daughter/Son
- Brother/Sister
- Father-in-law/Mother-in-law
- Son-in-law/daughter-in-law
- Brother-in-law/Sister-in-law
- Extended family (uncle/aunt/cousin/niece/nephew etc)
- No family relationship – guest

- Other

23. Is the principal applicant a male or a female?
Choose one response
- Male
- Female

24. How old is the principal applicant (in years)?

25. What is the highest level of education the principal applicant has completed?
Choose one response
- No formal schooling
- Some primary school
- Primary school completed
- Preparatory school completed
- Secondary school completed
- College/ University completed
- Don't Know
- Refused

26. Which governorate do you currently live in?
Choose one response
- Akkar
- Baalbek-El Hermel
- Beirut
- Bekaa
- El Nabatieh
- Mount Lebanon
- North
- South

27. Which district do you currently live in?
Choose one response
- Akkar
- Baalbek
- El Hermel
- Beirut
- Rachaya
- West Bekaa
- Zahle
- Bent Jbeil
- El Nabatieh
- Hasbaya
- Marjaayoun
- Aley
- Baabda
- Chouf
- El Meten
- Jbeil
- Kesrwane
- Bcharre
- El Batroun

- El Koura
- El Minieh'-Dennie
- Tripoli
- Zgharta
- Jezzine
- Saida
- Sour

28. Housing Type

Choose one response
- Apartment/house (finished)
- Single room in apartment/house
- Single room in other location (addition to home, in a workplace location such as garage or farm)
- Unfinished building or construction site
- Factory or warehouse
- Tent or temporary shelter built from other materials
- Prefab unit
- Collective center/shelter

Other

29. Occupancy Type

Choose one response
- Owned   If this response, jump to 31
- Rented (direct rent payment)   If this response, jump to 31
- Rented (in exchange of work)   If this response, jump to 31
- Hosted for free   If this response, jump to 31
- Rented/paid for by NGO/charity   If this response, jump to 31
- Squatting   If this response, jump to 31
- Other (specify)

30. Provide description for "other"

31. Excluding bathrooms/toilets, how many rooms does your residence have?

32. SECTION 3: HOUSEHOLD DEMOGRAPHICS
READ ALOUD: Now I will ask you some questions about your household. A household includes people who share a living space AND share meals and financial resources.
INTERVIEWER: Probe to ensure the respondent is correctly identifying the household. Households may include more than one UNHCR registration case. Extended family members that share the living space should only be considered household members if they regularly share meals and financial resources. Household members may or may not be biologically related. Short term visitors who stay for less than one month should not be counted household members.

33. How many people currently live in this household?

34. How many households live together in this residence?

35. How many people in the household are less than 5 years of age?

36. How many people in the household are age 5 to 17 (including age 17)?

37. How many people in the household are age 18 to 59 (including age 59)?

38. How many people in the household are age 60 or higher?

39. How many household members have chronic health conditions?
Chronic health conditions are deteriorations in health which require regular visits to a doctor or regular medication or treatment. For example, hypertension, kidney disease, neurological conditions like stroke or migraines, arthritis, cancer, or mental illness. Some people may have more than one chronic condition, but should be counted once.

40. How many household members have a disability or special needs (physical, hearing, visual, or mental disability)?
These are long term conditions which are not likely to change greatly in the next few months and limit normal function of a household member.

41. How many household members are in need of support to do daily activities due to other conditions (such as frailty or limitations due to old age)?

42. How many UNHCR registration cases are in this household?
A registration case is defined as a group of people that share the same UNHCR registration number (and are listed together on the same registration certificate or were recorded together when you received your token).

43.Please enter the first additional UNHCR case number in the household

44.Please enter the second additional UNHCR case number in the household

45. SECTION 4: HOUSEHOLD ECONOMY
READ ALOUD: Please estimate your household's expenses in the last month for the following expense categories (in LBP; enter '999' for not disclosed or '0' if household had no expense)
INTERVIEWER: if the household has multiple registration cases, remind the respondent to report this information for the entire household (those that live together and share financial resources/meals).

46. Housing / rent

47. Electricity or generator

48. Gas / Fuel / Wood (for cooking and heating only)

49. Water

50. Food

51. Consumable household items (toilet paper, soap, etc. Does not include food or larger purchases such as furniture)

52. Combined: Food and household items
NOTE: If respondent is unable to separate food and other household item expenditures, enter combined amount. If separate expenditures entered for each category, enter 0.

53. Telecommunication, internet, mobile subscription

54. Transportation

55. Health care (consultation fees, diagnostic testing, medicines)

56. Education (include transportation and supplies)

57. Clothes

58. Payment of debt

59. Any other large expenses that you have not mentioned? (Enter the amount of other large expenses in the last month)

60. Specify other large expenses

61. Were your household's expenditures in the past month similar to your usual expenditure levels?
Choose one response
- Yes If this response, jump to 63
- No, expenditures were higher than usual
- No, expenditures were lower than usual
- Don't Know If this response, jump to 63

62. If lower or higher, please explain:

63. Now I would like to ask a few questions about your households' spending related to health in the last month. For each question, please exclude spending using vouchers for health.
In the past month, has your household spent money on health (including medical consultations, diagnostic tests, medications, and/or transportation to receive these)?
Choose one response
- Yes
- No If this response, jump to 73
- Don't Know If this response, jump to 73

64. In the past month, how much has your household spent on medical consultations (in LBP)? Do not include the cost of medicines.
If the answer is "I don't know", please insert number 999.
If there were no expenses, enter "0"

65. In the past month, how much has your household spent on medical diagnostic tests (in LBP)?
If the answer is "I don't know", please insert number 999.
If there were no expenses, enter 0

66. In the past month, how much has your household spent on medications (in LBP)?
If the answer is "I don't know", please insert number 999.
If there were no expenses, enter 0

67. In the past month, how much has your household spent on transportation to receive medical care, diagnostic tests, or prescribed medication (in LBP)?
If the answer is "I don't know", please insert number 999.
If there were no expenses, enter 0

68. Were your household's expenditures on health in the past month similar to your usual expenditure levels?
Choose one response
- Yes If this response, jump to 71
- No, expenditures were higher than usual
- No, expenditures were lower than usual

- Don’t Know If this response, jump to 71

69. If lower or higher, please explain:

70. How much does your household spend on health in a usual month (including medical consultations, diagnostic tests, medications, and/or transportation to receive these)?
Enter ‘999’ for don't know

71. In the past month, did any organization give you money, either before or after the health expenses were incurred, that was intended to help cover medical costs?
Choose one response
- Yes
- No If this response, jump to 73
- Don’t Know If this response, jump to 73

72. How much money did they provide (in LBP)?

73. Please estimate your household's total income in the last month (in LBP). This may include income from a variety of sources such as salaries, pensions, investments or remittances. Do not include cash or vouchers received as humanitarian assistance.

74. How many household members worked in the last month?

75. From which type of employment is the households’ largest income source?
Choose one response
- Agriculture
- Construction
- Manufacturing
- Hotel/restaurant/sales
- Domestic service job
- Professional/salaried position
- Self-employment
- Transportation (eg: taxi driver)
- Doorman / building keeper
- Other

76. How much income came from this source in the last month (in LBP)?

77. Is this full-time or part-time employment?
Choose one response
- Full time
- Part time

78. Is this income regular?
Choose one response
- Regular (consistent/stable income)
- Irregular (varies daily/weekly)
- Seasonal (varies monthly/seasonally)

79. Did the household have employment income from any other sources?
Choose one response
- Yes

- No If this response, jump to 86

80. From which type of employment is the households' second largest income source?
Choose one response
- Agriculture
- Construction
- Manufacturing
- Hotel/restaurant/sales
- Domestic service job
- Professional/salaried position
- Self-employment
- Transportation (eg: taxi driver)
- Doorman / building keeper
- Other

81. How much income came from this source in the last month (in LBP)?

82. Is this full-time or part-time employment?
Choose one response
- Full time
- Part time

83. Is this income regular?
Choose one response
- Regular (consistent/stable income)
- Irregular (varies daily/weekly)
- Seasonal (varies monthly/seasonally)

84. Did the household have employment income from any other sources?
Choose one response
- Yes
- No If this response, jump to 86

85. From which other types of employment did the household earn income?
Choose all that apply
- Agriculture
- Construction
- Manufacturing
- Hotel/restaurant/sales
- Domestic service job
- Professional/salaried position
- Self-employment
- Transportation (eg: taxi driver)
- Doorman / building keeper
- Other

86. In the last month, did your household obtain income from any of the following sources?
If yes, enter monthly amount in LBP (total). If no, enter "0"

87. Profit from rental property you own (in Syria or elsewhere)

88. Remittances from family (abroad or employed elsewhere)

89. Gift from family/friend/other person

90. Loan

91. Asset Sales

92. Other
Enter the amount of income received from other sources.

93. Specify other income

94. SECTION 5: RECEIPT OF HUMANITARIAN ASSISTANCE

95. Since arriving to Lebanon, have you received regular monthly cash transfers from UNHCR? This is a regular transfer of the same amount provided every month to help households meet basic needs. Do not include intermittent transfers such as those provided in only in winter.
If yes, for how many years?
INTERVIEWER: If the household has come off and on UNHCR cash transfers, ask them to estimate each time period separately and then combine them to find the total length of time receiving UNHCR cash transfers. Only include cash transfers from UNHCR.
Choose one response
- Never received UNHCR assistance If this response, jump to 115
- < 6 months
- 6 months to < 1 year
- 1 year to < 2 years
- 2 years to < 3 years
- 3 or more years

96. Does your household currently receive monthly cash assistance from UNHCR?
Choose one response
- Yes
- No If this response, jump to 115
- Don't Know If this response, jump to 115

97. If yes, which case registration number(s) receive monthly cash assistance from UNHCR?
Enter case registration numbers, separating each with a comma (i.e. case#1,case#2,case#3....)

98. How much money did your household receive from UNHCR last month (in LBP)?

99. READ ALOUD: Now I would like to ask you about the use of UNHCR cash transfers. For each category that I read, please tell if you spend UNHCR cash on it or not. If yes, please tell me the expenditure amount for the last month. (in LBP; Record 0 for none, 999 for don't know)

100. Housing / rent

101. Electricity or generator

102. Gas / Fuel / Wood (for cooking and heating only)

103. Water

104. Consumable household items (toilet paper, soap, etc. Does not include larger purchases such as furniture)

105. Telecommunication, internet, mobile subscription

106. Food

107. Transportation

108. Medicines

109. Health care (consultation fees, diagnostic testing)

110. Education (include transportation and supplies)

111. Clothes

112. Payment of debt

113. Anything else?
Enter the amount of UNHCR cash transfers spent on other items.

114. Specify other cash spending

115. Since arriving to Lebanon, have you received regular monthly assistance from the World Food Programme? If yes, for how many years?
INTERVIEWER: If the household has come off and on WFP assistance, ask them to estimate each time period separately and then combine them to find the total length of time receiving WFP assistance. Only include assistance from WFP.
Choose one response
- Never received   If this response, jump to 119
- < 6 months
- 6 months to < 1 year
- 1 year to < 2 years
- 2 years to < 3 years
- 3 or more years

116. Does your household currently receive monthly cash or voucher assistance from the World Food Programme?
Choose one response
- Yes
- No   If this response, jump to 119
- Don’t Know   If this response, jump to 119

117. How much money did your household receive from WFP last month (in LBP)?

118. Is the assistance provided as a store credit or cash collected at an ATM?
Choose one response
- E-card
- Cash transfer

119. Since arriving to Lebanon, have you received regular monthly cash or voucher assistance from any other organization?
Include only cash transfers that are provided on a monthly basis for the same amount.

If yes, for how many years? If multiple sources, record the longest.
INTERVIEWER: If the household has come off and on other cash/voucher assistance, ask them to estimate each time period separately and then combine them to find the total length of time receiving other cash/voucher assistance.
Choose one response
- Never received   If this response, jump to 124
- < 6 months
- 6 months to < 1 year
- 1 year to < 2 years
- 2 years to < 3 years
- 3 or more years

120. Does your household currently receive regular monthly cash or voucher assistance from any other organization? Include only cash transfers that are provided on a monthly basis for the same amount.
Choose one response
- Yes
- No   If this response, jump to 124
- Don't Know   If this response, jump to 124

121. Which organization(s) provides assistance?

122. Is the assistance unrestricted or is it intended to be used for certain types of expenses only? Include only transfers that are provided on a monthly basis for the same amount.
Choose all that apply
- Unrestricted cash
- Cash for rent
- Voucher for rent
- Cash for education
- Cash for shelter
- Cash for health
- Other cash (specify)

123. In total, how much did your household receive in cash/voucher assistance last month from all sources other than UNHCR and WFP (LBP)?

124. Has your household received any other type of cash or voucher assistance in the past 3 months? Include any cash/voucher assistance that was received from humanitarian organizations on an irregular basis (most often a single transfer). For example, cash for child birth or winterization.
Choose one response
- Yes
- No   If this response, jump to 128
- Don't Know   If this response, jump to 128

125. From which organization(s)?

126. How much cash/voucher assistance was received, in total, from all organizations, in the past 3 months (in LBP)? Enter the combine amount from all sources. If necessary, help the respondent by listing transfers from each source separately; then record the total.

127. What was the assistance provided for?

128. Did your household receive any in-kind assistance in the past 3 months?
Choose one response

- Yes
- No If this response, jump to 131
- Don’t Know If this response, jump to 131

129. What type(s) of assistance were received?
Choose all that apply
- Accommodation
- Shelter materials/repairs
- Utilities (fuel, electricity, water)
- Household items / Clothing
- Food
- Medication
- Health services
- Education
- Business/livelihood inputs
- Other (specify)

130. Provide description for "other". Leave blank if "other" was not selected in previous question.

131. SECTION 6: ASSET SALES AND BORROWING

132. In the past 3 months, have you had to sell any assets in order to pay for routine household expenses?
Choose one response
- Yes
- No If this response, jump to 139
- Don’t Know If this response, jump to 139

133. What types of assets did you sell?
Choose all that apply
- Jewelry or gold
- Household assets (appliances, electronics, etc.)
- Vehicle (car, motorcycle)
- Property (land, house)
- Productive assets (personal or business items used for income generation)
- Other

134. How much money did your household earn from asset sales in the past 3 months (in LBP)?

135. What did you use the majority of money from asset sales for?
If money was spent on multiple categories, record the category where most of the money was spent.
Choose one response
- Rent If this response, jump to 137
- Food If this response, jump to 137
- Medication If this response, jump to 137
- Health service If this response, jump to 137
- Education If this response, jump to 137
- Business/livelihood If this response, jump to 137
- Shelter repairs If this response, jump to 137
- Utilities (fuel, electricity, water) If this response, jump to 137
- Household items / Clothing If this response, jump to 137
- Other (specify)

136. Provide description for "other"

137. Did you spend money from asset sales on anything else?
Choose all that apply
- No
- Rent
- Food
- Medication
- Health service
- Education
- Business/livelihood
- Shelter repairs
- Utilities (fuel, electricity, water)
- Household items / Clothing
- Other (specify)

138. Provide description for "other". Leave blank if "other" was not selected in previous question.

139. In the past 3 months, have you had to borrow any money in order to pay for routine household expenses?
Choose one response
- Yes
- No If this response, jump to 146
- Don't Know If this response, jump to 146

140. From whom have you borrowed? (Note: if borrowing from more than one source, select the source from which the most was borrowed)
Choose all that apply
- Family
- Friends
- Informal lender
- Microfinance organization
- Bank
- Shawish
- Store (bought food on credit)
- Local associations/charity (for example, a mosque)
- Landlord
- Stranger
- Other ی

141. How much money did your household borrow in the past 3 months (in LBP)?
If necessary, help the respondent by listing the amount borrowed from each source separately; then record the total.

142. What did you use the majority of borrowed money for?
If money was spent on multiple categories, record the category where most of the money was spent.
Choose one response
- Rent If this response, jump to 144
- Food If this response, jump to 144
- Medication If this response, jump to 144
- Health service If this response, jump to 144
- Education If this response, jump to 144
- Business/livelihood If this response, jump to 144
- Shelter repairs If this response, jump to 144

- Utilities (fuel, electricity, water)   If this response, jump to 144
- Household items / Clothing   If this response, jump to 144
- Other (specify)

143. Provide description for "other"

144. Did you spend borrowed money on anything else?
Choose all that apply
- No
- Rent
- Food
- Medication
- Health service
- Education
- Business/livelihood
- Shelter repairs
- Utilities (fuel, electricity, water)
- Household items / Clothing
- Other (specify)

145. Provide description for "other". Leave blank if "other" was not selected in previous question.

146. How much money is currently owed by your household (in LBP)? Include money borrowed within the past 3 months in addition to any other outstanding debt.

147. SECTION 7: CHILD HEALTH CARE SEEKING
Read aloud: Now I would like to ask some questions about the last time a child (under 18 years old) in your household needed health care.

148. DO NOT READ ALOUD: Is there anyone under 18 years of age in this household?
Choose one response
- Yes
- No   If this response, jump to 216

149. When was the last time a CHILD (under 18 years old) in your household had a health problem that you felt needed medical care?   Only include health problems that occurred while in Lebanon, even if you did not take the child to a doctor, pharmacy or other medical provider.
INTERVIEWER: If more than two children were sick at the same time as the caregiver which child was the sickest and answer the following set of questions only for the sickest child.
Choose one response
- Less than two weeks ago
- Between 2 weeks and less than 1 month ago
- Between 1 month and less than 3 months ago
- Between 3 months and less than 6 months ago
- Between 6 months and less than 1 year ago
- More than 1 year ago   If this response, jump to 216
- Never needed care in Lebanon   If this response, jump to 216
- Don't know   If this response, jump to 216

150. How old is this child currently?
NOTE: record age in years. If the child is 0-11 months old, you should write "0". If the child is 12-23 months old, you should write "1". If the child is 24-35 months old, you should write "2". etc.

151. Child sex
Choose one response
- Male
- Female

152. Which reason best describes why health care was needed? (if more than one, record the most concerning)
Choose one response
- Fever If this response, jump to 154
- Diarrhea If this response, jump to 154
- Respiratory problem such as cough, cold, flu or difficulty breathing (not chronic) If this response, jump to 154
- Asthma If this response, jump to 154
- Injury If this response, jump to 154
- Dental care If this response, jump to 154
- Behavioral or emotional problem If this response, jump to 154
- Eye problem If this response, jump to 154
- Ear problem If this response, jump to 154
- Skin problem If this response, jump to 154
- Worms If this response, jump to 154
- Other (specify)

153. Provide description for "other"

154. Did the child receive medical attention for the condition?
Choose one response
- Yes, sought and received
- No, did not seek care If this response, jump to 214
- No, sought but no care received If this response, jump to 214
- Don't Know If this response, jump to 216

155. Where did (child's name) first seek care for the condition?
Choose one response
- Primary health center
- Private clinic
- Home based provider (Syrian)
- Home based provider (Lebanese)
- Hospital
- Mobile Medical Unit (MMU)
- Pharmacy
- Home visit (provider consulted in the respondent's home)
- Other

156. What was the reason for choosing to seek care from that provider? (Record primary reason)
Choose one response
- Free consultation services
- Low cost consultation services
- Closest to place of residence
- No other facility nearby
- Like staff/quality/family doctor
- Free or reduced cost medications provided
- Free or reduced diagnostic tests provided
- Availability of needed services

- Medical emergency
- Short waiting time / easy to get an appointment
- Not sick enough to take to health facility
- Did not know any other providers
- Other (specify)

157. Provide description for other. Leave blank if other was not selected in the previous question.

158. What type of hospital was used?
Choose one response
- Private hospital
- Public hospital
- Don't know

159. What type of visit was this?
Choose one response
- Emergency room visit (where people receive urgent care when they are very ill) If this response, jump to 166
- Outpatient visit If this response, jump to 166
- Inpatient admission (when you are given a bed at the hospital and stay overnight or longer)

160. How many nights was the hospital stay?

161. Did UNHCR or any other organization assist in paying for some of the fees?
Choose one response
- Yes, UNHCR
- Yes, other organization
- Yes, both
- No, none
- Don't Know

162. Did you/your household have to pay for any of the cost yourself?
Choose one response
- Yes
- No If this response, jump to 166
- Don't Know If this response, jump to 166

163. How much did your household pay for the hospital visit (in LBP, excluding the amount paid by any organization on your behalf)?

164. Did you pay for the hospital visit on credit?
Choose one response
- Yes
- No If this response, jump to 166

165. How much is still owed (in LBP)?

166. Were any medicines prescribed at this visit?
Choose one response
- Yes, provided at the health facility If this response, jump to 168
- Yes, but purchased at another location
- Yes, medicine prescribed but not obtained
- No medicine prescribed If this response, jump to 168

167. Why were you not able to get the medicines that were prescribed? (Record the primary reason)
Choose one response
- Medication was out of stock at the facility
- Medication was not available at the facility
- Medication was too expensive
- Other

168. Did your household have to pay for the visit?
Payment may include consultation fees for the provider, diagnostic testing, medicines, or transportation to get to/from the facility.
Choose one response
- Yes
- No If this response, jump to 176
- Don't Know If this response, jump to 176

169. What was the total amount that your household paid at the facility (LBP)?

170. How much money did your household pay to the provider for consultation fees (LBP)?

171. How much money did your household pay for diagnostic testing (LBP)?

172. How much money did your household pay for medicines (at the facility, do not include medicines purchased elsewhere; in LBP)?

173. Of the medicines you obtained at the health facility, did you get any on credit?
Choose one response
- Yes
- No If this response, jump to 175

174. How much is still owed (in LBP)?

175. How much money did your household pay for transportation to/from the facility (in LBP)?

176. Were there any diagnostic tests, treatments, or medicines that were recommended but that you could not afford?
Choose one response
- Yes
- No If this response, jump to 179

177. What was recommended that you could not afford?
Choose all that apply
- Diagnostic tests
- Medication
- Specialist Consultation
- Medical Equipment
- Other

178. What was the total cost of recommended tests/treatments/medicines that were not obtained because of cost (in LBP)?

179. Was (name) referred to another facility or provider for care?
Choose one response

- Yes, received referral care
- Yes, but did not receive referral care
- No If this response, jump to 203
- Don't Know If this response, jump to 203

180. What was (name) referred for?
Record primary reason if multiple reasons are provided.
Choose one response
- Diagnostic or laboratory test
- Specialist consultation
- Curative intervention (specific medication, surgery, procedure)
- Other

181. Where was the child referred?
Choose one response
- Mobile Medical Unit (MMU) If this response, jump to 183
- Primary health center If this response, jump to 183
- Private clinic If this response, jump to 183
- Medical center If this response, jump to 183
- Hospital
- Other If this response, jump to 183

182. What type of hospital was used?
Choose one response
- Private hospital
- Public hospital
- Don't know

183. What type of visit was this?
Choose one response
- Emergency room visit (where people receive urgent care when they are very ill) If this response, jump to 190
- Outpatient visit If this response, jump to 190
- Inpatient admission (when you are given a bed at the hospital and stay overnight or longer)

184. How many nights was the hospital stay?

185. Did UNHCR or any other organization assist in paying for some of the fees?
Choose one response
- Yes, UNHCR
- Yes, other organization
- Yes, both
- No, none
- Don't Know

186. Did you/your household have to pay for any of the cost yourself?
Choose one response
- Yes
- No If this response, jump to 190
- Don't Know If this response, jump to 190

187. How much did your household pay for the hospital visit (in LBP, excluding the amount paid by any organization on your behalf)?

188. Did you pay for the hospital visit on credit?
Choose one response
- Yes
- No   If this response, jump to 190

189. How much is still owed (in LBP)?

190. Were any medicines prescribed at the referral visit?
Choose one response
- Yes, provided at the health facility   If this response, jump to 192
- Yes, but purchased at another location
- Yes, medicine prescribed but not obtained
- No medicine prescribed   If this response, jump to 192

191. Why were you not able to get the medicines that were prescribed? (Record the primary reason)
Choose one response
- Medication was out of stock at the referral facility
- Medication was not available at the referral facility
- Medication was too expensive
- Other

192. Did your household have to pay for the referral provider visit?
Payment may include consultation fees for the provider, diagnostic testing, medicines, or transportation to get to/from the facility.
Choose one response
- Yes
- No   If this response, jump to 200
- Don't Know   If this response, jump to 200

193. What was the total amount that your household paid at the referral facility (LBP)?

194. How much money did your household pay to the referral provider for consultation fees (LBP)?

195. How much money did your household pay at the referral facility for diagnostic testing (LBP)?

196. How much money did your household pay for medicines at the referral facility (do not include medicines purchased elsewhere; in LBP)?

197. Of the medicines you obtained at the referral facility, did you get any on credit?
Choose one response
- Yes
- No   If this response, jump to 199

198. How much is still owed (in LBP)?

199. How much money did your household pay for transportation to/from the referral facility (in LBP)?

200. Were there any diagnostic tests, treatments, or medicines that were recommended at the referral facility but that you could not afford?
Choose one response
- Yes

- No	If this response, jump to 203

201. What was recommended that you could not afford?
Choose all that apply
- Diagnostic tests
- Medication
- Specialist Consultation
- Medical Equipment
- Other

202. What was the total cost of recommended tests/treatments/medicines that were not obtained because of cost (in LBP)?

203. Were any medications prescribed for the child's illness that were not obtained at the facilities where you received care?
Choose one response
- Yes
- No	If this response, jump to 216

204. Did you attempt to obtain these medications elsewhere?
Choose one response
- Yes
- No	If this response, jump to 208

205. Where did you attempt to obtain the prescribed medications?
Choose all that apply
- At another facility (different from where the doctor was seen)
- Private pharmacy
- Syria
- Via a local contact/fixer
Other

206. Provide description for "other". Leave blank if "other" was not selected in previous question.

207. Were you able to get all of the medications that were prescribed?
Choose one response
- Yes, all of the medications	If this response, jump to 210
- Yes, some of the medications
- No
- Don’t Know	If this response, jump to 216

208. Why were you not able to get all of the medicines that were prescribed? (Record the primary reason)
Choose one response
- Household could not afford the medication
- Did not know where to get the medication
- Medication was out of stock at the facility
- Medication was out of stock at private pharmacy
- Symptoms improved/child began to feel better
- Household chose a different treatment
- Household decided the medicines were not needed
- Purchasing medication was not a priority
- Too far to travel to obtain medication

- Other (specify)

209. Provide description for "other". Leave blank if "other" was not selected in previous question.

210. Did your household have to pay for the medications received outside the facilities where you received care?
Choose one response
- Yes
- No   If this response, jump to 216

211. How much money did your household pay for medicines outside the facility where you received care (do not include medicines purchased at the care facility; in LBP)?

212. Of the medicines you obtained outside the health facilities where care was received, did you get any on credit?
Choose one response
- Yes
- No   If this response, jump to 216

213. How much is still owed (in LBP)?

214. What was the main reason for not seeking medical care for the child's health condition? (Record one, Primary reason)
Choose one response
- Could not afford provider costs   If this response, jump to 216
- Distance or lack of transportation   If this response, jump to 216
- Could not afford transportation costs   If this response, jump to 216
- Provider's equipment or drugs are inadequate   If this response, jump to 216
- Disliked previous visit(s) (attitude)   If this response, jump to 216
- Disliked previous visit(s) (long waiting time)   If this response, jump to 216
- Disliked previous visit(s) (quality of care)   If this response, jump to 216
- Could not take time off work or had other commitments   If this response, jump to 216
- Didn't know where to go   If this response, jump to 216
- Family decided care should not be sought (no male permission)   If this response, jump to 216
- Security concerns and/or check points   If this response, jump to 216
- Appointment scheduled / still waiting   If this response, jump to 216
- Other (specify)

215. Provide description for "other"

216. SECTION 8: HEALTH CARE SEEKING FOR CHRONIC CONDITIONS
Now I am going to ask some questions about chronic health conditions in your household. Chronic health conditions are those which last for long periods of time, often many years, and may never be cured. Examples of chronic conditions include hypertension or high blood pressure, diabetes, heart problems and long term respiratory diseases, although there are many others. Include only adults 18 years and older.

217. How many of your household members have been diagnosed with a chronic health condition such as diabetes or hypertension?

218. I would like to ask some questions about each person in your household that has been diagnosed with a chronic health condition.
Subform name : H3SD_R2HC_MPC_Enrollment_Lebanon_NCDSub_v3
Subform keyword : NCD

219. SECTION 9: ACUTE ILLNESS
Read aloud: Now I would like to ask some questions about the last time an adult (over 18 years old) in your household needed health care for an acute illness.

220. When was the last time an adult (over 18 years old) in your household been acutely ill with a health problem where they needed medical care? Include health problems that occurred, even if the individual did not receive medical care from a doctor, pharmacy, or other medical provider. Do not include chronic medical conditions. INTERVIEWER: If more than two acute illness occurred within the past month, ask the respondent which was most severe and answer questions only for that occurrence.
Choose one response
- Less than two weeks ago
- Between 2 weeks and less than 1 month ago
- Between 1 month and less than 3 months ago
- Between 3 months and less than 6 months ago
- Between 6 months and less than 1 year ago
- More than 1 year ago If this response, jump to 287
- Never needed care in Lebanon If this response, jump to 287
- Don't know If this response, jump to 287

221. How old is (name) currently (in years)?

222. (Name)'s sex
Choose one response
- Male
- Female

223. Which reason best describes why health care was needed? (if more than one, record the most concerning)
Choose one response
- Injury If this response, jump to 225
- Infection or communicable disease (including cough, cold, flu, and diarrhoea) If this response, jump to 225
- Dental Care If this response, jump to 225
- Skin problem If this response, jump to 225
- Emotional or mental health If this response, jump to 225
- Complications related to pregnancy If this response, jump to 225
- Gynecological problem If this response, jump to 225
- Asthma/breathing difficulty If this response, jump to 225
- Other (specify)

224. Provide description for "other"

225. Was medical attention received for the condition?
Choose one response
- Yes, sought and received
- No, did not seek care If this response, jump to 285
- No, sought but no care received If this response, jump to 285
- Don’t Know If this response, jump to 287

226. Where did (name) first seek care for the condition?
Choose one response
- Primary health center
- Private clinic
- Home based provider (Syrian)

- Home based provider (Lebanese)
- Hospital
- Mobile Medical Unit (MMU)
- Pharmacy
- Home visit (provider consulted in the respondent’s home)
- Other

227. What was the reason for choosing to seek care from that provider? (Record primary reason)
Choose one response
- Free consultation services
- Low cost consultation services
- Closest to place of residence
- No other facility nearby
- Like staff/quality/family doctor
- Free or reduced cost medications provided
- Free or reduced diagnostic tests provided
- Availability of needed services
- Medical emergency
- Short waiting time / easy to get an appointment
- Not sick enough to take to health facility
- Did not know any other providers
- Other (specify)

228. Provide description for other. Leave blank if other was not selected in the previous question.

229. What type of hospital was used?
Choose one response
- Private hospital
- Public hospital
- Don’t know

230. What type of visit was this?
Choose one response
- Emergency room visit (where people receive urgent care when they are very ill) If this response, jump to 237
- Outpatient visit If this response, jump to 237
- Inpatient admission (when you are given a bed at the hospital and stay overnight or longer)

231. How many nights was the hospital stay?

232. Did UNHCR or any other organization assist in paying for some of the fees?
Choose one response
- Yes, UNHCR
- Yes, other organization
- Yes, both
- No, none
- Don’t Know

233. Did you/your household have to pay for any of the cost yourself?
Choose one response
- Yes
- No If this response, jump to 237
- Don’t Know If this response, jump to 237

234. How much did your household pay for the hospital visit (in LBP, excluding the amount paid by any organization on your behalf)?

235. Did you pay for the hospital visit on credit?
Choose one response
- Yes
- No If this response, jump to 237

236. How much is still owed (in LBP)?

237. Were any medicines prescribed at this visit?
Choose one response
- Yes, provided at the health facility If this response, jump to 239
- Yes, but purchased at another location
- Yes, medicine prescribed but not obtained
- No medicine prescribed If this response, jump to 239

238. Why were you not able to get all of the prescribed medicines at the health facility? (Record the primary reason)
Choose one response
- Medication was out of stock at the facility
- Medication was not available at the facility
- Medication was too expensive
- Other

239. Did your household have to pay for the visit?
Payment may include consultation fees for the provider, diagnostic testing, medicines, or transportation to get to/from the facility.
Choose one response
- Yes
- No If this response, jump to 247
- Don't Know If this response, jump to 247

240. What was the total amount that your household paid at the facility (LBP)?

241. How much money did your household pay to the provider for consultation fees (LBP)?

242. How much money did your household pay for diagnostic testing (LBP)?

243. How much money did your household pay for medicines (at the facility, do not include medicines purchased elsewhere; in LBP)?

244. Of the medicines you obtained at the health facility, did you get any on credit?
Choose one response
- Yes
- No If this response, jump to 246

245. How much is still owed (in LBP)?

246. How much money did your household pay for transportation to/from the facility (in LBP)?

247. Were there any diagnostic tests, treatments, or medicines that were recommended but that you could not

afford?
Choose one response
- Yes
- No If this response, jump to 250

248. What was recommended that you could not afford?
Choose all that apply
- Diagnostic tests
- Medication
- Specialist Consultation
- Medical Equipment
- Other

249. What was the total cost of recommended tests/treatments/medicines that were not obtained because of cost (in LBP)?

250. Was (name) referred to another facility or provider for care?
Choose one response
- Yes, received referral care
- Yes, but did not receive referral care
- No If this response, jump to 274
- Don't Know If this response, jump to 274

251. What was (name) referred for?
Record primary reason if multiple reasons are provided.
Choose one response
- Diagnostic or laboratory test
- Specialist consultation
- Curative intervention (specific medication, surgery, procedure)
- Other

252. Where was (name) referred?
Choose one response
- Mobile Medical Unit (MMU) If this response, jump to 254
- Primary health center If this response, jump to 254
- Private clinic If this response, jump to 254
- Medical center If this response, jump to 254
- Hospital
- Other If this response, jump to 254

253. What type of hospital was used?
Choose one response
- Private hospital
- Public hospital
- Don't know

254. What type of visit was this?
Choose one response
- Emergency room visit (where people receive urgent care when they are very ill) If this response, jump to 261
- Outpatient visit If this response, jump to 261
- Inpatient admission (when you are given a bed at the hospital and stay overnight or longer)

255. How many nights was the hospital stay?

256. Did UNHCR or any other organization assist in paying for some of the fees?
Choose one response
- Yes, UNHCR
- Yes, other organization
- Yes, both
- No, none
- Don't Know

257. Did you/your household have to pay for any of the cost yourself?
Choose one response
- Yes
- No If this response, jump to 261
- Don't Know If this response, jump to 261

258. How much did your household pay for the hospital visit (in LBP, excluding the amount paid by any organization on your behalf)?

259. Did you pay for the hospital visit on credit?
Choose one response
- Yes
- No If this response, jump to 261

260. How much is still owed (in LBP)?

261. Were any medicines prescribed at the referral visit?
Choose one response
- Yes, provided at the health facility If this response, jump to 263
- Yes, but purchased at another location
- Yes, medicine prescribed but not obtained
- No medicine prescribed If this response, jump to 263

262. Why were you not able to get all of the prescribed medicines at the referral facility? (Record the primary reason)
Choose one response
- Medication was out of stock at the referral facility
- Medication was not available at the referral facility
- Medication was too expensive
- Other

263. Did your household have to pay for the referral provider visit?
Payment may include consultation fees for the provider, diagnostic testing, medicines, or transportation to get to/from the facility.
Choose one response
- Yes
- No If this response, jump to 271
- Don't Know If this response, jump to 271

264. What was the total amount that your household paid at the referral facility (LBP)?

265. How much money did your household pay to the referral provider for consultation fees (LBP)?

266. How much money did your household pay at the referral facility for diagnostic testing (LBP)?

267. How much money did your household pay for medicines at the referral facility (do not include medicines purchased elsewhere; in LBP)?

268. Of the medicines you obtained at the referral facility, did you get any on credit?
Choose one response
- Yes
- No If this response, jump to 270

269. How much is still owed (in LBP)?

270. How much money did your household pay for transportation to/from the facility (in LBP)?

271. Were there any diagnostic tests, treatments, or medicines that were recommended at the referral facility but that you could not afford?
Choose one response
- Yes
- No If this response, jump to 274

272. What was recommended that you could not afford?
Choose all that apply
- Diagnostic tests
- Medication
- Specialist Consultation
- Medical Equipment
- Other

273. What was the total cost of recommended tests/treatments/medicines that were not obtained because of cost (in LBP)?

274. Were any medications prescribed for (name)'s illness that were not obtained at the facilities where care was received?
Choose one response
- Yes
- No If this response, jump to 287

275. Did you attempt to obtain these medications elsewhere?
Choose one response
- Yes
- No If this response, jump to 279

276. Where did you attempt to obtain the prescribed medications?
Choose all that apply

- At another facility (different from where the doctor was seen)
- Private pharmacy
- Syria
- Via a local contact/fixer
Other

277. Provide description for "other". Leave blank if "other" was not selected in previous question.

278. Were you able to get all of the medications that were prescribed?
Choose one response
- Yes, all of the medications If this response, jump to 281
- Yes, some of the medications
- No
- Don't Know If this response, jump to 287

279. Why were you not able to get all of the medicines that were prescribed? (Record the primary reason)
Choose one response
- Household could not afford the medication
- Did not know where to get the medication
- Medication was out of stock at the facility
- Medication was out of stock at private pharmacy
- Symptoms improved/(name) began to feel better
- Household chose a different treatment
- Household decided the medicines were not needed
- Purchasing medication was not a priority
- Too far to travel to obtain medication
- Other (specify)

280. Provide description for "other". Leave blank if "other" was not selected in previous question.

281. Did your household have to pay for the medications received outside the facilities where you received care?
Choose one response
- Yes
- No If this response, jump to 287

282. How much money did your household pay for medicines outside the facility where you received care (do not include medicines purchased at the care facility; in LBP/JD)?

283. Of the medicines you obtained outside the health facilities where care was received, did you get any on credit?
Choose one response
- Yes
- No If this response, jump to 287

284. How much is still owed (in LBP)?

285. What was the main reason for not seeking medical care for the health condition? (Record one, primary reason)
Choose one response
- Could not afford provider costs If this response, jump to 287
- Distance or lack of transportation If this response, jump to 287
- Could not afford transportation costs If this response, jump to 287
- Provider's equipment or drugs are inadequate If this response, jump to 287
- Disliked previous visit(s) (attitude) If this response, jump to 287
- Disliked previous visit(s) (long waiting time) If this response, jump to 287
- Disliked previous visit(s) (quality of care) If this response, jump to 287
- Could not take time off work or had other commitments If this response, jump to 287
- Didn't know where to go If this response, jump to 287
- Family decided care should not be sought (no male permission) If this response, jump to 287
- Security concerns and/or check points If this response, jump to 287
- Appointment scheduled / still waiting If this response, jump to 287

- Other (specify)

286. Provide description for "other"

287. SECTION 10: SEXUAL AND REPRODUCTIVE HEALTH

288. Are any women in the household currently pregnant or have they given birth in the past six months?
Choose one response
- No If this response, jump to 302
- Currently pregnant If this response, jump to 289
- Recently gave birth If this response, jump to 290
- Miscarriage If this response, jump to 302

289. When do you expect the baby to be born?

290. Month of birth
Choose one response
- January
- February
- March
- April
- May
- June
- July
- August
- September
- October
- November
- December

291. Year of Birth
Choose one response
- 2017
- 2018
- 2019

292. In what type of facility did [name] deliver the baby?
Choose one response
- Public hospital
- Private hospital or clinic
- Home
- Other

293. What was the type of delivery?
Choose one response
- Vaginal Normal
- Caesarean section
- Don’t Know

294. Did [name] have to pay anything herself for this delivery?
Choose one response
- Yes

- No If this response, jump to 296

295. Approximately how much did they pay (in LBP)?

296. Did [name] receive any financial support from UNHCR or an NGO to help pay for the delivery costs?
Choose one response
- Yes
- No If this response, jump to 300

297. Was this a payment to the health facility or a cash transfer or reimbursement to [name] directly?
Choose one response
- Health facility If this response, jump to 300
- Individual cash transfer If this response, jump to 299
- Other (specify)

298. Provide description for "other"

299. How much was the transfer/reimbursement (in LBP)?

300. Did you know that UNHCR provides support for delivery at hospital?
Choose one response
- Yes
- No If this response, jump to 302

301. Did you know how to access this support?
Choose one response
- Yes
- No

302. SECTION 11: HOUSEHOLD HEALTH CARE ACCESS PERCEPTIONS
I am going to read you five statements about access to medical care. Please tell me if you agree or disagree with each statement.

303. My household is able to get medical care whenever we need it.
Choose one response
- Strongly agree
- Somewhat agree
- Somewhat disagree
- Strongly disagree
- Non applicable or no opinion

304. My household has access to the medical specialists we need.
Choose one response
- Strongly agree
- Somewhat agree
- Somewhat disagree
- Strongly disagree
- Non applicable or no opinion

305. My household can always afford medical care.
Choose one response
- Strongly agree

- Somewhat agree
- Somewhat disagree
- Strongly disagree
- Non applicable or no opinion

306. My household can always afford the medications we need.
Choose one response
- Strongly agree
- Somewhat agree
- Somewhat disagree
- Strongly disagree
- Non applicable or no opinion

307. My household receives enough health information.
Choose one response
- Strongly agree
- Somewhat agree
- Somewhat disagree
- Strongly disagree
- Non applicable or no opinion

308. INTERVIEWER: This is the end of the questionnaire. Please thank the participant for their time.

309. Please write any additional comment about the interview that study coordinators need to know. If possible, please enter comment(s) in English.

310. Did you complete the full interview?
Choose one response
- Yes
- No, but I have arranged to call the respondent back to complete the interview
- No, respondent refused to participate
- No, wrong phone number provided
- No, patient did not meet eligibility criteria

Subform: H3SD_R2HC_MPC_Enrollment_Lebanon_NCDSub_v3
65 Questions

===

1. How old is (name) currently (in years)?

2. (Name)'s sex
Choose one response
- Male
- Female

3. What chronic medical conditions has (name) been diagnosed with?
Choose all that apply
- Hypertension
- Diabetes
- Arthritis
- Chronic respiratory disease
- Cardiovascular disease
- Cancer
- Other (specify)

4. Provide description for “other”. Leave blank if "other" was not selected in previous question.

5. When was the last time that (name) saw a doctor for their chronic medical condition while in Lebanon?
Choose one response
- Less than 1 month If this response, jump to 8
- Between 1 month and less than 3 months ago If this response, jump to 8
- Between 3 months and less than 6 months ago If this response, jump to 8
- Between 6 months and less than 1 year ago If this response, jump to 8
- More than 1 year ago If this response, jump to 54
- Never needed/sought care in Lebanon
- Don’t Know If this response, jump to 54

6. What was the main reason for not seeking medical care for (name)'s chronic condition? (Record one, Primary reason)
Choose one response
- Could not afford provider costs If this response, jump to 54
- Distance or lack of transportation If this response, jump to 54
- Could not afford transportation costs If this response, jump to 54
- Provider’s equipment or drugs are inadequate If this response, jump to 54
- Disliked previous visit(s) (attitude) If this response, jump to 54
- Disliked previous visit(s) (long waiting time) If this response, jump to 54
- Disliked previous visit(s) (quality of care) If this response, jump to 54
- Could not take time off work or had other commitments If this response, jump to 54
- Didn't know where to go If this response, jump to 54
- Family decided care should not be sought (no male permission) If this response, jump to 54
- Security concerns and/or check points If this response, jump to 54
- Appointment scheduled / still waiting If this response, jump to 54
- Not sick enough to go to health facility If this response, jump to 54
- Other (specify)

7. Provide description for "other"

8. For the last time (name) needed care for chronic medical conditions in Lebanon, where did (name) first go?
Choose one response
- Primary health center If this response, jump to 10
- Private clinic If this response, jump to 10
- Home based provider (Syrian) If this response, jump to 10
- Home based provider (Lebanese) If this response, jump to 10
- Hospital If this response, jump to 10
- Mobile Medical Unit (MMU) If this response, jump to 10
- Pharmacy If this response, jump to 10
- Home visit (provider consulted in the respondent's home) If this response, jump to 10
- Other (specify)

9. Provide description for "other"

10. What was the reason for choosing to seek care from that provider? (Record primary reason)
Choose one response
- Free consultation services
- Low cost consultation services
- Closest to place of residence
- No other facility nearby
- Like staff/quality/family doctor
- Free or reduced cost medications provided
- Free or reduced diagnostic tests provided
- Availability of needed services
- Medical emergency
- Short waiting time / easy to get an appointment
- Not sick enough to take to health facility
- Did not know any other providers
- Other (specify)

11. Provide description for other. Leave blank if other was not selected in the previous question.

12. What type of hospital was used?
Choose one response
- Private hospital
- Public hospital
- Don't know

13. What type of visit was this?
Choose one response
- Emergency room visit (where people receive urgent care when they are very ill If this response, jump to 20
- Outpatient visit If this response, jump to 20
- Inpatient admission (when you are given a bed at the hospital and stay overnight or longer)

14. How many nights was the hospital stay?

15. Did UNHCR or any other organization assist in paying for some of the fees?
Choose one response
- Yes, UNHCR
- Yes, other organization
- Yes, both

- No, none
- Don’t Know

16. Did you/your household have to pay for any of the cost yourself?
Choose one response
- Yes
- No If this response, jump to 27
- Don't Know If this response, jump to 27

17. How much did your household pay for the hospital visit (in LBP, excluding the amount paid by any organization on your behalf)?

18. Did you pay for the hospital visit on credit?
Choose one response
- Yes
- No If this response, jump to 27

19. How much is still owed (in LBP)?

20. Did your household have to pay for the provider visit?
Payment may include consultation fees for the provider, diagnostic testing, medicines, or transportation to get to/from the facility.
Choose one response
- Yes, paid some of the cost
- Yes, paid all of the cost If this response, jump to 22
- No If this response, jump to 27

21. Who paid the other portion of the cost ?

22. What was the total amount that your household paid at the facility? (LBP) Do not include amounts that were paid on your behalf or reimbursed.

23. How much money did your household pay to the provider for consultation fees (in LBP)?

24. How much money did your household pay for diagnostic testing (LBP)?

25. How much money did your household pay for medicines (at the facility, do not include medicines purchased elsewhere; in LBP)?

26. How much money did your household pay for transportation to/from the facility (in LBP)?

27. Were there any diagnostic tests, treatments, or medicines that were recommended but that you could not afford?
Choose one response
- Yes
- No If this response, jump to 30

28. What was recommended that you could not afford?
Choose all that apply
- Diagnostic tests
- Medication
- Specialist Consultation
- Medical Equipment

- Other

29. What was the total cost of recommended tests/treatments/medicines that were not obtained because of cost (in LBP)?

30. Was (name) referred to another facility or provider for care?
Choose one response
- Yes, received referral care / نعم، تقى خدمات اتحوي
- Yes, but did not receive referral care
- No / ا If this response, jump to 50
- Don't Know If this response, jump to 50

31. What was (name) referred for?
Choose one response
- Lab tests / فحوصات مخبرية
- Diagnostic test / وسائ تشخيص
- Specialist Consultation
- Curative intervention (specific medication, surgery, procedure) / تدخ عاجي - أدوية ، جراحة، عاج مختص
- Other

32. Where was (name) referred?
Choose one response
- Mobile Medical Unit (MMU) If this response, jump to 34
- Primary health center If this response, jump to 34
- Private clinic If this response, jump to 34
- Medical center / مركز صحي If this response, jump to 34
- Hospital
- Other If this response, jump to 34

33. What type of hospital was used?
Choose one response
- Private hospital
- Public hospital
- Don't know

34. What type of visit was this?
Choose one response
- Emergency room visit (where people receive urgent care when they are very ill) / زيارة غرفة اطوارئ)اماكن اذي يتقى فيه الأشخاص ارعاية اعاجة إذا كانوا شديدي امرض(If this response, jump to 41
- Outpatient visit If this response, jump to 41
- Inpatient admission (when you are given a bed at the hospital and stay overnight or longer)

35. How many nights was the hospital stay?

36. Did UNHCR or any other organization assist in paying for some of the fees?
Choose one response
- Yes, UNHCR
- Yes, other organization
- Yes, both
- No, none
- Don't Know

37. Did you/your household have to pay for any of the cost yourself?
Choose one response
- Yes
- No If this response, jump to 47
- Don't Know If this response, jump to 47

38. How much did your household pay for the hospital visit (in LBP, excluding the amount paid by any organization on your behalf)?

39. Did you pay for the hospital visit on credit?
Choose one response
- Yes
- No If this response, jump to 47

40. How much is still owed (in LBP)?

41. Did your household have to pay for the referral provider visit?
Payment may include consultation fees for the provider, diagnostic testing, medicines, or transportation to get to/from the facility.
Choose one response
- Yes
- No If this response, jump to 47
- Don't Know If this response, jump to 47

42. What was the total amount that your household paid at the referral facility (LBP)?

43. How much money did your household pay to the referral provider for consultation fees (in LBP)?

44. How much money did your household pay at the referral facility for diagnostic testing (in LBP)?

45. How much money did your household pay for medicines referral facility (do not include medicines purchased elsewhere; in LBP)?

46. How much money did your household pay for transportation to/from the facility (in LBP)?

47. Were there any diagnostic tests, treatments, or medicines that were recommended at the referral facility but that you could not afford?
Choose one response
- Yes
- No If this response, jump to 50

48. What was recommended that you could not afford?
Choose all that apply
- Diagnostic tests
- Medication
- Specialist Consultation
- Medical Equipment / امعدات/أأجهزة اطبية
- Other

49. What was the total cost of recommended tests/treatments/medicines that were not obtained because of cost (in LBP)?

50. How many times has (name) visited a General Practitioner (GP) in the last 6 months?

51. How many times has (name) seen another doctor/specialist (not GP) in the last 6 months?

52. How many times has (name) consulted a pharmacist in the last 6 months?

53. How many visits to a hospital for chronic medical conditions has (name) had in the last 6 months?

54. Has (name) been prescribed, by a doctor/pharmacist, medicines for their chronic condition(s) (in Lebanon or in Syria)?
Choose one response
- Yes, by a doctor in Syria
- Yes, by a pharmacist in Syria
- Yes, by a doctor in Lebanon
- Yes, by a pharmacist in Lebanon
- No If this response, jump to 65

55. How many medications is (name) currently prescribed for chronic conditions?

56. In the past month, how often has (name) taken the medications prescribed for chronic conditions?
Choose one response
- Always / دائما
- Most of the time (>50% of doses taken as prescribed)
- Some of the time (<50% of doses taken as prescribed)
- Never (did not take medication in the past month)

57. Does the household face difficulties obtaining medication prescribed for (name)?
Choose one response
- Yes, very often
- Yes, sometimes
- Never If this response, jump to 60

58. What difficulties did you face in obtaining medication? (Record primary difficulty)
Choose all that apply
- Medication was out of stock at the facility
- Household could not afford the medication
- Did not know where to get the medication
- Difficulty finding a pharmacy with my medication
- Too far to travel to obtain medication
- Did not like what was available
- Other (specify)

59. Provide description for "other". Leave blank if "other" was not selected in previous question.

60. Where does (name) most often obtain medication?
Choose one response
- At the facility where the doctor was seen If this response, jump to 62
- At another facility (different from where the doctor was seen) If this response, jump to 62
- Private pharmacy If this response, jump to 62
- Syria If this response, jump to 62
- Via a local contact/fixer If this response, jump to 62
- Other (specify)

61. Provide description for "other"

62. On average, how much does the household pay for (name)'s medications per month (in LBP)?
If medication received in longer supply, calculate average monthly price (i.e. for 3-month supply divide cost by 3).

63. Have you received any medicines for (name)'s condition on credit?

Choose one response

- Yes
- No If this response, jump to 65

64. How much is still owed (in LBP)?

65. End of chronic condition questions for this individual
