# Supplementary material for: Multi-purpose cash transfers and health among vulnerable Syrian refugees in Lebanon: a prospective cohort study
Source: BMC Public Health. 2021 Jun 19;21:1176. doi: 10.1186/s12889-021-11196-8 (PMC8214292; doi:10.1186/s12889-021-11196-8)
Supplement: Supplementary file 3 — Additional file 3. Health Care-Seeking and Medicines for Household Member Illness at Baseline and Endline. Description: Baseline and endline descriptive analyses of care-seeking outcomes by group. [file 12889_2021_11196_MOESM3_ESM.pdf]

Health Care-Seeking and Medicines for Household Member Illness at Baseline and Endline

	BASELINE			ENDLINE		
	**MPC HHs** (N=168) % (95% CI)	**Control HHs** (N=375) % (95% CI)	P value	**MPC HHs** (N=168) % (95% CI)	**Control HHs** (N=375) % (95% CI)	P value
**Most Recent Childhood Illness**						
**Access to Health Services and Medicines**						
Sought and received medical care	98.4% (96.1,100%)	92.8% (89.8,95.8%)	0.133	98.0% (95.8,100%)	91.6% (88.4,94.8%)	0.062
Able to obtain prescribed medications	99.2% (97.5,100%)	98.0% (96.3,99.8%)	0.417	99.3% (98.0,100%)	97.7% (95.9,99.5%)	0.230
**Health Service Utilization**						
Outpatient visit	83.6% (76.9,90.3%)	82.7% (78.1,87.2%)		94.6% (90.9,98.3%)	88.2% (84.4,92.1%)	
Emergency room visit	13.9% (7.7,20.2%)	16.2% (11.8,20.7%)	0.521	2.0% (0.3,4.3%)	3.7% (1.4,5.9%)	0.101
Hospital admission	2.5% (0.3,5.2%)	1.1% (0.1,2.4%)		3.4% (0.4,6.3%)	8.1% (4.8,11.3%)	
**Cost as Barrier to Health**						
Medical care not sought because of cost	100% --	94.7% (83.7,100%)	0.740	66.7% (76.8,210.1%)	83.3% (67.3,99.4%)	0.484
All needed care not received due to cost	6.6% (2.1,11.0%)	10.3% (6.7,14.0%)	0.230	45.9% (37.8,54.1%)	37.9% (32.1,43.7%)	0.107
**Most Recent Adult Acute Illness**						
**Access to Health Services and Medicines**						
Sought and received medical care	88.3% (81.0,95.7%)	90.6% (86.2,95.1%)	0.202	92.6% (87.8,97.3%)	85.4% (80.8,90.0%)	0.064
Able to obtain prescribed medications	100% --	97.9% (95.5,100%)	0.229	97.1% (93.9,100%)	98.9% (97.4,100%)	0.261
**Health Service Utilization**						
Outpatient visit	86.8% (78.5,95.0%)	87.7% (82.5,93.0%)		91.1% (85.7,96.4%)	94.3% (91.0,97.6%)	
Emergency room visit	13.2% (5.0,21.5%)	11.6% (6.5,16.7%)	0.761	4.5% (0.6,8.3%)	2.6% (0.3,4.9%)	0.549
Hospital admission	0.0% --	0.6% (0.6,1.9%)		4.5% (0.6,8.3%)	3.1% (0.6,5.6%)	
**Cost as Barrier to Health**						
Medical care not sought because of cost	100% --	100% --	--	66.7% (28.2,100%)	87.9% (76.1,99.6%)	0.130
All needed care not received due to cost	19.1% (9.5,28.7%)	19.4% (13.1,25.6%)	0.967	41.1% (31.8,50.3%)	35.2% (28.4,42.0%)	0.310
**Adult Chronic Illness**						
**Access to Health Services and Medicines**						
Sought and received medical care	93.5% (84.4,102.7%)	90.8% (84.1,97.4%)	0.641	95.8% (91.1,100%)	95.2% (91.9,98.5%)	0.833
Ever faced difficulties obtaining medication	72.4% (55.1,89.7%)	61.4% (49.7,73.1%)	0.298	72.5% (61.7,83.3%)	73.1% (66.2,80.1%)	0.918
**Health Service Utilization**						
General practitioner visit(s)	76.9% (59.6,94.3%)	71.0% (59.3,82.6%)	0.567	56.7% (43.8,69.6%)	55.7% (47.4,64.0%)	0.901
Specialist visit(s)	77.8% (61.0,94.5%)	77.4% (66.7,88.1%)	0.970	70.0% (58.1,81.9%)	63.4% (55.4,71.4%)	0.366
Hospital visit(s)	14.8% (0.5,29.1%)	32.3% (20.3,44.2%)	0.088	15.0% (5.7,24.3%)	32.4% (24.6,40.2%)	0.011
**Cost as Barrier to Health**						
Medical care not sought because of cost	0.0% --	100% --	---	100% --	100% --	---
All needed care not received due to cost	33.3% (14.3,52.3%)	23.8% (13.0,34.6%)	0.349	39.3% (26.7,52.0%)	51.4% (43.1,59.7%)	0.114
Cannot afford medication	61.3% (43.1,79.5%)	46.1% (34.6,57.5%)	0.153	63.9% (52.5,75.3%)	65.3% (58.0,72.6%)	0.837
