# Supplementary material for: Multi-purpose cash transfers and health among vulnerable Syrian refugees in Lebanon: a prospective cohort study
Source: BMC Public Health. 2021 Jun 19;21:1176. doi: 10.1186/s12889-021-11196-8 (PMC8214292; doi:10.1186/s12889-021-11196-8)
Supplement: Supplementary file 4 — Additional file 4. Health Expenditures for Most Recent Child, Adult Acute, and Adult Chronic Illness Care and in the Preceding Month (USD) at Baseline and Endline. Description: Baseline and endline descriptive analyses of health expenditure outcomes by group. [file 12889_2021_11196_MOESM4_ESM.pdf]

## Health Expenditures for Most Recent Child, Adult Acute, and Adult Chronic Illness Care and in the Preceding Month (USD [a]) at Baseline and Endline

| | BASELINE | | | | | ENDLINE | | | | |
|---|---|---|---|---|---|---|---|---|---|---|
| | MPC HHs (N=173) | | Control HHs (N=444) | | | MPC HHs (N=168) | | Control HHs (N=375) | | |
| | Mdn | Mean (95% CI) | Mdn | Mean (95% CI) | P value | Mdn | Mean (95% CI) | Mdn | Mean (95% CI) | P value |
| **Most Recent Childhood Illness** | | | | | | | | | | |
| **Health Facility Payments for OP Care [b]** | | n=119 | | *n=268* | | | *n=143* | | *n=250* | |
| Any payment for outpatient care at facility | -- | 85.7% (79.3,92.1%) | -- | 86.6% (82.5,90.7%) | 0.822 | -- | 88.8% (83.6,94.0%) | -- | 87.2% (83.0,91.4%) | 0.639 |
| Total paid at facility for visit (all HHs) | 13 | 32.0 (18.7,45.3) | 13 | 29.1 (20.7,37.6) | 0.715 | 15 | 32.3 (17.1,47.5) | 12 | 30.4 (19.3,41.6) | 0.845 |
| **Medication Costs at Pharmacy/Elsewhere** | | n=122 | | *n=271* | | | *n=148* | | *n=272* | |
| Any payment for medication outside facility | -- | 52.5% (43.5,61.4%) | -- | 48.0% (42.0,54.0%) | 0.410 | -- | 68% (60.7,75.8%) | -- | 64.3% (58.6,70.1%) | 0.421 |
| Total paid for medication (all HHs) | 7 | 15.4 (11.0,19.7) | 0 | 11.4 (9.0,13.9) | 0.095 | 10 | 17.8 (12.6,23.0) | 11 | 18.4 (15.1,21.7) | 0.852 |
| **Total Amount Paid for Illness [c]** | | n=119 | | *n=268* | | | *n=143* | | *n=250* | |
| Any expense for most recent illness | -- | 84.9% (78.3,91.4%) | -- | 81.7% (77.1,86.4%) | 0.449 | -- | 89.5% (84.4,94.6%) | -- | 88.4% (84.4,92.4%) | 0.737 |
| Total cost for most recent illness (all HHs) | **22** | **44.1 (29.8,58.5)** | **20** | **29.1 (23.9,34.2)** | **0.016** | 23 | 45.6 (29.0,62.3) | 23 | 43.9 (32.2,55.7) | 0.868 |
| **Most Recent Acute Adult Illness** | | | | | | | | | | |
| **Health Facility Payments for OP Care [b]** | | n=68 | | *n=154* | | | *n=107* | | *n=187* | |
| Any payment for outpatient care at facility | -- | 94.1% (88.4,99.9%) | -- | 87.0% (81.6,92.4%) | 0.116 | -- | 82.2% (74.9,89.6%) | -- | 87.7% (82.9,92.5%) | 0.198 |
| Total paid at facility for visit (all HHs) | 20 | 34.7 (22.1,47.3) | 15 | 44.1 (20.6,67.7) | 0.609 | **13** | **47.1 (21.2,72.9)** | **13** | **24.3 (18.7,29.9)** | **0.031** |
| **Medication Costs at Pharmacy/Elsewhere** | | n=68 | | *n=154* | | | *n=107* | | *n=187* | |
| Any payment for medication outside facility | -- | 61.8% (49.9,73.6%) | -- | 52.6% (44.6,60.6%) | 0.205 | -- | 55.1% (45.6,64.7%) | -- | 54.5% (47.3,61.7%) | 0.921 |
| Total paid for medication (all HHs) | 13 | 18.9 (13.7,24.1) | 4 | 16.8 (12.5,21.1) | 0.576 | 7 | 17.8 (12.4,23.2) | 6 | 17.8 (12.9,22.6) | 0.999 |
| **Total Amount Paid for Illness [c]** | | n=68 | | *n=154* | | | *n=107* | | *n=187* | |
| Any expense for most recent illness | -- | 89.7% (82.3,97.1%) | -- | 82.5% (76.4,88.5%) | 0.167 | -- | 77.6% (69.5,85.6%) | -- | 81.8% (76.2,87.4%) | 0.379 |
| Total cost for most recent illness (all HHs) | 39 | 45.2 (34.8,55.7) | 20 | 44.4 (30.5,58.4) | 0.942 | 23 | 42.8 (27.4,58.2) | 25 | 38.3 (31.1,45.5) | 0.554 |
| **Most Recent Adult Chronic Illness Visit** | | | | | | | | | | |
| **Health Facility Payments for OP Care [b]** | | n=27 | | n=63 | | | n=61 | | n=142 | |
| Any payment for outpatient care at facility | -- | 70.4% (52.0,88.8%) | -- | 68.3% (56.4,80.1%) | 0.842 | -- | 75.4% (64.3,86.5%) | -- | 78.9% (72.1,85.7%) | 0.586 |
| Total paid at facility for visit (all HHs) | 5 | 39.1 (14.0,64.1) | 13 | 31.3 (16.4,46.2) | 0.576 | 13 | 36.5 (22.8,50.3) | 20 | 40.1 (30.9,49.4) | 0.670 |
| **Average Monthly Medication Costs** | | n=31 | | n=76 | | | n=72 | | n=167 | |
| Any regular (monthly) medication costs | -- | 64.5% (46.7,82.4%) | -- | 69.7% (59.2,80.3%) | 0.599 | -- | 69.4% (58.5,80.3%) | -- | 74.9% (68.2,81.5%) | 0.387 |
| Average monthly medication costs (all cases) | 33 | 39.1 (22.5,55.7) | 19 | 31.8 (20.9,42.8) | 0.470 | 21 | 30.6 (22.9,38.3) | 20 | 34.7 (27.1,42.3) | 0.528 |
| **Routine Spending on Health** | | *n=173* | | *n=444* | | | *n=168* | | *n=375* | |
| Health Expenditures (past month) [d] | **53** | **88 (70.5,104.7)** | **37** | **60.9 (51.0,70.7)** | **0.006** | **66** | **102.2 (83.3,121.0)** | **60** | **82.5 (72.7,92.3)** | **0.045** |
| Sold assets to pay for health (past 3 months) | -- | 3.5% (0.7,6.2%) | -- | 3.2% (1.5,4.8%) | 0.843 | -- | 6.0% (2.3,9.6%) | -- | 10.1% (7.1,13.2%) | 0.113 |
| Borrowed to pay for health (past 3 months) | **--** | **23.7% (17.3,30.1%)** | **--** | **15.8% (12.4,19.2%)** | **0.021** | **--** | **66.1% (58.8,73.3%)** | **--** | **50.7% (45.6,55.8%)** | **0.001** |

HH = households; Mdn = median; OP = outpatient. [a] reported in USD; exchange rate: 1507.5 LBP = 1 USD; [b] includes consultation fees, diagnostic testing and medications obtained at health facility during the initial visit to health facility, hospital outpatient department, or emergency room (without overnight stay); [c] includes health facility payments for outpatient care and medications purchased at pharmacies outside health facilities (does not include referrals, in-patient care and transportation); [d] at facilities and for medication
